# Surface Avidity of Anionic Polypeptide Coatings on Layer‐by‐Layer Nanoparticles Target Cancer‐Associated Amino Acid Transporters

**DOI:** 10.1002/anie.202519203

**Published:** 2025-12-22

**Authors:** Ivan S. Pires, Margaret M. Billingsley, Ezra Gordon, Andrew J. Pickering, Eva Cai, Gonzalo J. Esparza, Mae L. Pryor, Alexander D. Stoneman, Aidan Kindopp, Darrell J. Irvine, Paula T. Hammond

**Affiliations:** ^1^ Koch Institute for Integrative Cancer Research Massachusetts Institute of Technology 500 Main Street Cambridge Massachusetts 02139 USA; ^2^ Department of Chemical Engineering Massachusetts Institute of Technology 21 Ames Street Cambridge Massachusetts 02139 USA; ^3^ Harvard‐MIT Health Sciences and Technology Massachusetts Institute of Technology 77 Massachusetts Ave Cambridge Massachusetts 02139 USA; ^4^ Department of Biological Engineering Massachusetts Institute of Technology 25 Ames Street Cambridge Massachusetts 02139 USA; ^5^ Department of Materials Science and Engineering Massachusetts Institute of Technology 182 Memorial Dr Cambridge Massachusetts 02139 USA; ^6^ Ragon Institute of MGH, MIT and Harvard University 600 Main St Cambridge Massachusetts 02139 USA; ^7^ Howard Hughes Medical Institute 4000 Jones Bridge Rd Chevy Chase Maryland 20815 USA

**Keywords:** Cancer targeting, Drug delivery, Layer‐by‐layer, Nanoparticles, Polymer coating

## Abstract

Tumor‐targeted drug delivery enhances therapeutic efficacy while minimizing toxicity. Layer‐by‐layer nanoparticles (LbL‐NPs) coated with anionic polypeptides selectively bind to cancer cells, though the mechanisms have been unclear. Here, we integrated in silico and in vitro approaches—including gene expression analysis, receptor inhibition, and AI‐based protein modeling—to show that poly(L‐glutamate) (PLE)‐coated LbL‐NPs bind with high avidity to SLC1A5, a glutamine transporter overexpressed in cancer. We also discovered that PLE clusters SLC1A5 on the cell membrane, promoting prolonged cell surface retention. Poly(L‐aspartate) (PLD)‐coated NPs similarly bind SLC1A5 but also interact with faster internalizing transporters of anionic amino acids. Correlation analyses across cancer cell lines confirmed a strong link between transporter expression and nanoparticle (NP) association. These findings demonstrate that dense glutamate or aspartate presentation through electrostatically adsorbed polypeptides enables selective targeting of overexpressed transporters, providing a mechanistic framework for receptor‐targeted delivery that leverages metabolic characteristics of a range of solid tumor types.

## Graphical abstract

Electrostatically layered nanoparticles (NPs) coated with anionic polypeptides selectively target cancer cells by engaging amino acid transporters. Poly(L‐glutamate) (PLE) coatings cluster the glutamine transporter SLC1A5, prolonging NP surface retention, while poly(L‐aspartate) (PLD) coatings also bind SLC1A3, potentially promoting faster internalization. This mechanistic framework reveals how multivalent polypeptide presentation enables receptor‐specific NP delivery across diverse tumor types.

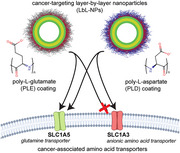

## Introduction

Nanoparticles (NPs) are promising vehicles for drug delivery due, in part, to their ability to modulate drug bioavailability and pharmacokinetics.^[^ ^1^, ^2^ ^]^ A central challenge in cancer nanomedicine is achieving selective delivery of therapeutics to malignant cells while minimizing off‐target toxicity in healthy tissues. Early strategies relied on the enhanced permeability and retention (EPR) effect, which exploits the leaky vasculature of tumors to promote NP accumulation.^[^ ^3^, ^4^ ^]^ However, the EPR effect has demonstrated inconsistent efficacy across tumor types and limited clinical translation. To address these limitations, alternative models such as active transport and retention (ATR) have recently been proposed to better explain and predict NP accumulation in tumors.^[^ ^5^ ^]^ Beyond non‐specific targeting, functionalizing NPs with antibodies, peptides, or small molecules enables more selective delivery by engaging specific cell‐surface receptors.^[^ ^4^ ^]^ Despite its promise, this approach faces challenges, including the scarcity of truly cancer‐specific targets, tumor heterogeneity, and the immunogenicity or manufacturing complexity of targeting ligands. These limitations have prompted growing interest in alternative mechanisms for tumor‐specific NP binding that do not rely on conventional ligand‐receptor paradigms. Among these, a promising method to modulate NP properties and enable cell‐specific targeting is through the established electrostatic layer‐by‐layer (LbL) technique for functionalizing NP surfaces.^[^ ^6^, ^7^, ^8^ ^]^

We previously reported that coating the surface of NPs with charged polypeptides composed of poly‐L‐aspartate (PLD) and poly‐L‐glutamate (PLE) enhanced their affinity and specificity toward multiple cancer cells both in vitro and in vivo and impacted NP internalization rates.^[^ ^7^, ^9^, ^10^ ^]^ This unique targeting strategy makes layer‐by‐layer nanoparticles (LbL‐NPs) of interest as potentially broadly applicable cancer‐targeting delivery vehicles. Indeed, we have successfully used PLE‐ and PLD‐coated LbL‐NPs to target therapeutics delivery to tumor tissues in multiple murine cancer models^[^ ^10^, ^11^, ^12^, ^13^ ^]^ However, unlike some LbL‐NPs with high cancer cell affinity—such as hyaluronic acid (HA)‐coated particles that target CD44 receptors and promote rapid receptor‐mediated endocytosis—the receptor(s) mediating specific binding of these anionic polypeptide coatings to cancer cells have remained unclear.^[^ ^7^ ^]^

One potential mechanism of the cancer‐specific association of these polypeptide coatings is through amino acid transporters. Cancer cells often become dependent on certain amino acids such as glutamine or aspartate,^[^ ^14^, ^15^, ^16^, ^17^ ^]^ and to sustain this intracellular amino acid pool requirement, the cancer cells modulate a network of redundant transmembrane proteins that mediate cellular amino acid transport.^[^ ^18^ ^]^ Here, we explore the increased expression of amino acid transporters as a potential mechanism underlying the specificity of polypeptide LbL‐NPs binding to cancer cells. Through a combination of experimental approaches, data analytics, and artificial intelligence protein interaction modeling, we demonstrate that LbL film assembly allows for high avidity presentation of amino acid side chains that interact with amino acid transporters overexpressed by cancer cells. Further, we show how PLE cell membrane retention is enhanced by cell surface receptor cluster formation. Last, we find that cellular gene expression of amino acid transporters correlates with efficient LbL‐NP association while demonstrating that PLD NPs further interact with different amino acid transporters than PLE NPs, which may contribute to differences observed in their cellular trafficking. Together, these findings provide insights into the mechanisms of anionic polypeptide‐based targeting for NP delivery across cancer cells.

## Results and Discussion

### Polypeptide Presentation and Avidity Regulate Cancer Cell Binding Affinity

**Figure 1:**
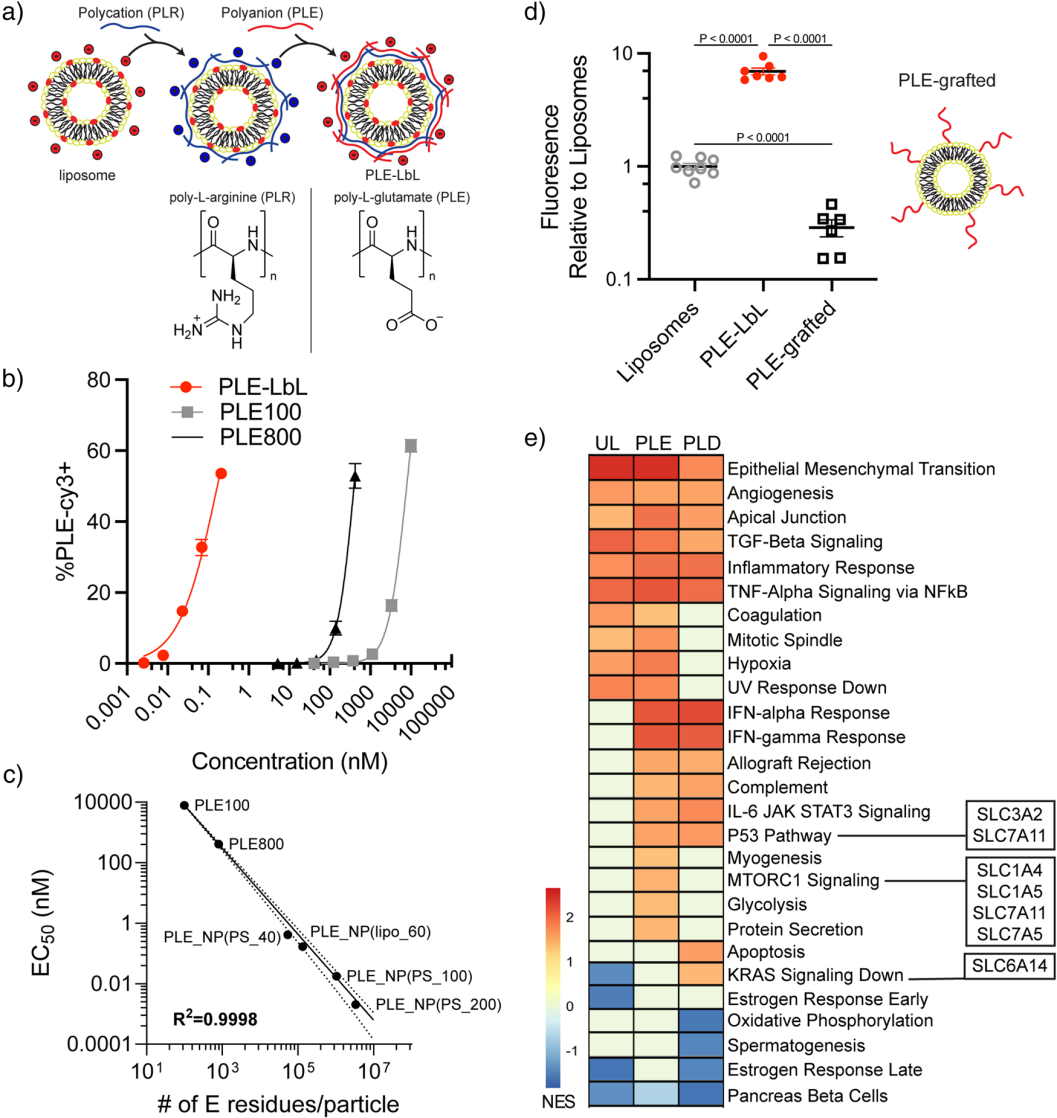
Polypeptide LbL films enable high‐affinity NP binding to cancer cells. a) Schematic of LbL surface modification in which polymers of alternating charge are electrostatically layered onto the NP surface, with structures of the polycation and poly amino acids used in this figure shown below. b) Fluorescently tagged PLE was dosed at varying concentrations to HM‐1 cells for 4 h either in the LbL‐film on NP (PLE_100_‐NP) or as free polymers at a 100 or 800 degree of polymerization, and flow cytometry was used to measure the percentage of PLE + cells (mean ± s.d.). c) Relationship between estimated number of glutamate (E) residues per particle or per molecule for free polymer and the derived EC_50_ from the experiment in b) and EC_50_ of NP + cells dosed with PLE‐coated carboxylate‐modified PS NPs of varying diameters (40, 100, and 200 nm). d) Fluorescently labeled NPs were dosed to HM‐1 cells in vitro at 1 µg mL^−1^. Four hours after dosing, cells were washed, and NP fluorescence associated with cells was measured on a plate reader. Shown are the normalized fluorescence readings relative to a UL negatively charged liposome (mean ± s.d.). e) Data from NanoPrism^[^ ^27^ ^]^ was used to rank cell lines based on their association with each NP (UL, PLE, or PLD), and the gene expression of the top 100 and bottom 100 cell lines was then compared via GSEA against the hallmark gene sets. The heatmap shows the normalized enrichment score (NES) of significant gene sets (FDR *q*‐val < 0.05) for any of the three NP groups, with positive values indicating these gene sets were enriched in the cell lines that corresponded with high NP association. Genes for relevant amino acid transporters are noted next to their corresponding gene sets. Statistical comparison in d) was performed using one‐way analysis of variance (ANOVA) with Tukey's multiple‐comparisons test. Data are representative of at least two independent experiments with *n* = 3 technical replicates per group.

Cancer cell‐targeting LbL‐NPs are formed by sequential adsorption of oppositely charged polymers onto a charged NP surface.^[^ ^7^, ^13^, ^19^ ^]^ This established technique of electrostatic polymer layering has been broadly utilized across nanotechnology applications to generate functional materials.^[^ ^8^, ^20^, ^21^, ^22^, ^23^, ^24^, ^25^ ^]^ In the case of poly(L‐glutamate) (PLE)‐coated LbL‐NPs, a bilayer film composed of positively‐charged poly(L‐arginine) (PLR) and negatively‐charged PLE assembled onto a negatively‐charged liposome core is sufficient to generate high affinity binding to cancer cell surfaces in vivo (Figure 1a).^[^ ^13^, ^26^ ^]^ We first sought to determine if PLE has an intrinsic high‐affinity for cancer cells and if incorporation of this polypeptide into the LbL film coating the NP surface plays an important role in its binding activity. The ovarian cancer cell line OV2944‐HM1 (HM‐1) was incubated with two PLE polymers at varying degrees of polymerization (PLE_100_ or PLE_800_) or with LbL‐NPs coated with PLE_100_ polypeptide (PLE_100_‐NPs), and cell association was measured by flow cytometry. PLE_100_ was used as the standard polymer size for LbL assembly as in our prior ovarian cancer‐targeted PLE‐NPs^[^ ^7^, ^10^, ^13^, ^26^, ^27^, ^28^ ^]^ because its shorter chain length reduces particle bridging during layering^[^ ^29^ ^]^ and facilitates renal clearance upon release.^[^ ^30^ ^]^ While increasing the polymer chain length did allow for a ∼9‐fold increase in EC_50_, PLE_100_‐NPs exhibited a much higher apparent affinity of binding, with an EC_50_ 50 000‐fold lower than free PLE_100_, demonstrating a major effect of presentation of the PLE from NP surfaces on cell association (Figure 1b).

To better understand how incorporating PLE into an LbL‐NP led to this increase in association, we explored the effect of glutamate residue multivalency on cancer cell affinity. We evaluated PLE_100_ and PLE_800_ free polymers, 60 nm liposomal PLE_100_‐NPs, and fluorescently‐labeled carboxylate‐modified polystyrene (PS) NPs of varying sizes (diameters of 40, 100, and 200 nm) coated with PLR followed by a PLE_100_ outer layer. The varying of diameters allows us to compare a range of glutamate (E) residues per particle, and their resulting affinity toward HM‐1 cells was quantified. Comparing the estimated number of glutamate (E) residues per particle (determined from maximum polymer loading based on zeta potential plateau^[^ ^28^ ^]^) or free polymer molecule to the observed cellular apparent affinity yielded a clear log–log relationship suggesting that the high surface avidity of glutamate residues from the LbL film plays a role in the high‐affinity interactions (Figure 1c). This association may also be influenced by NP characteristics such as relative stiffness or size, but the observed trend holds across the PS NPs, free polymers, and liposomes explored here.^[^ ^27^, ^31^, ^32^, ^33^ ^]^

To understand if the mode of PLE presentation from NPs is important for its high cellular affinity, we next evaluated the binding of fluorescently‐labeled unmodified liposomes, liposomes coated with PLE_100_ either via an electrostatically adsorbed LbL film, or liposomes bearing PLE_100_ covalently end‐grafted to the liposome surface (Figure 1d), and measured cell‐associated fluorescence after 4 h of in vitro incubation. We maximized the amount of grafted PLE polymers on the NPs to yield liposomes of ∼100 nm in size and similar negative surface charge (Figure S1a–c). Increasing the grafting density beyond 0.15 weight equivalents of PLE to total lipids led to disc or micelle formation due to charge and steric repulsion.^[^ ^34^ ^]^ While PLE‐LbL coating increased NP binding ∼10‐fold compared to unlayered (UL) liposomes, grafted PLE polymers unexpectedly reduced binding by a similar magnitude (Figure 1d). This may suggest that grafted polypeptides create extended brush‐like layers that sterically inhibit rather than promote cell binding.^[^ ^35^, ^36^ ^]^ Chain conformations of electrostatically layered polymers in LbL films are quite different and present as loops and trains bound on the surface.^[^ ^36^ ^]^ These results suggest that the nature of polypeptide presentation from the NP surface is critical for determining cellular interactions. Notably, while it is possible to generate monolayer PLE‐coated NPs onto cationic liposomes (Figure S1d,e), these did not confer increased cellular association over UL NPs (Figure S1f) likely due to both increased non‐specific association of underlying cationic NPs and low film stability of monolayer‐coated NPs.

Across these evaluations of cell association, the cellular affinity of PLE was highest when presented via the LbL NP platform and directly correlated with glutamate residue valency per particle. We sought to understand what transcriptional profiles promote binding of the polyvalent glutamate‐presenting PLE‐NPs to the surface of cancer cells by mining our previously published nanoPRISM dataset, which contains data from high‐throughput screens measuring the association of various NPs with or without LbL coatings to a library of 488 human cancer cell lines.^[^ ^27^ ^]^ Specifically, we looked to compare the performance of UL liposomes with liposomes layered with PLE. In addition to PLE LbL‐NPs, we also evaluated the binding of poly‐L‐aspartate (PLD)‐coated NPs given the close similarity between these anionic polypeptides and their cancer cell targeting properties demonstrated in previous work (Figure S1g).^[^ ^7^ ^]^ Within each NP group—UL, PLE, and PLD—we compared the gene expression of the 100 cell lines exhibiting the highest NP association with the gene expression of the 100 cell lines exhibiting the lowest NP association. We then rank‐ordered the genes based on the highest *p*‐value adjusted fold‐difference in expression and performed gene set enrichment analysis (GSEA) against hallmark gene sets (Figure 1e). From this analysis, many gene signatures were identified. For example, epithelial‐to‐mesenchymal (EMT) transition was enriched for high association with all three NP groups, which may be due to higher overall cellular activity and NP uptake. Moreover, consistent with prior experiments, STAT3 signaling was a significant hit for increasing the association of PLE‐liposomes.^[^ ^37^ ^]^

In further analysis, we focused on identifying cell surface‐expressed features that mediate the interaction between the anionic, glutamate‐rich PLE coating and cancer cells. Given that PLE is composed entirely of glutamate residues and that we observed increased cellular association with increasing glutamate presentation, we hypothesized that specific amino acid transporters could play a role in mediating this interaction. We searched for relevant amino acid transporters among the top enriched gene sets corresponding to high‐association cell lines for PLE‐NPs, PLD‐NPs, or both. This effort identified SLC1A4, SLC1A5, and SLC7A5 for PLE‐enriched gene sets; SLC6A14 for PLD‐enriched gene sets; and SLC3A2 and SLC7A11 for both PLE‐ and PLD‐NP enriched gene sets (Figure 1e). Notably, all the identified genes are direct transporters or promote glutamine uptake with highly interconnected functions. For example, SLC3A2 and SLC7A5 encode for proteins that heterodimerize to achieve functional expression in the plasma membrane, SLC1A5 and SLC7A5 often act in opposition to balance intracellular glutamine concentration, and SLC7A11 increases SLC1A5 glutamine uptake by exporting intracellular glutamate.^[^ ^38^, ^39^, ^40^ ^]^ Additionally, a number of these transporters are commonly overexpressed in cancer cells, including SLC1A5, SLC7A5, and SLC7A11.^[^ ^41^, ^42^, ^43^ ^]^ These results suggested an influence of these different amino acid transporters—especially glutamine transporters—on LbL‐NP/cell association.

### Glutamine Transport Inhibitors Block PLE‐NP Binding to Cancer Cells

**Figure 2:**
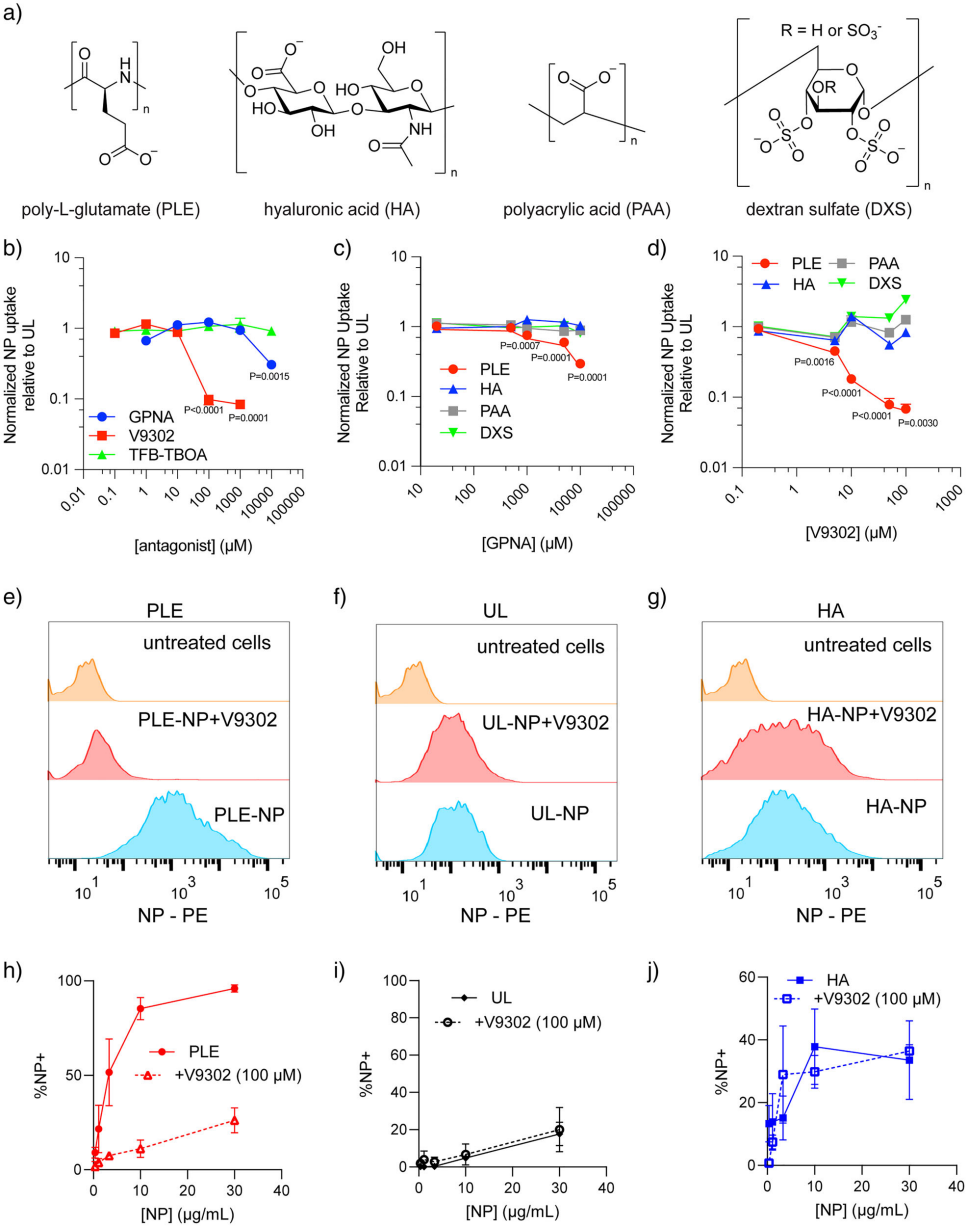
Association of PLE‐coated NPs is blocked with glutamine transport inhibitors. a) Chemical structures of the outer layer polyanions used in this work. b)–d) HM‐1 cells were treated with varying concentrations of amino acid transport inhibitors for 15 min before NP dosing at 50 µg mL^−1^ b) or 25 µg mL^−1^ c), d) per well. Four b) or two c), d) hours after NP dosing, wells were washed and total NP fluorescence was measured via plate reader to determine NP association. Shown are the normalized NP association of PLE‐coated NPs relative to UL at different inhibitor concentrations of GPNA, TFB‐TBOA, and V9302 (mean ± s.d., b), and the normalized NP association of various outer layer LbL‐coated NPs relative to UL at different inhibitor concentrations of GPNA (mean ± s.d., c) or V9302 (mean ± s.d., d). e)–j) HM‐1 cells were treated with 100 µM of V9302 for 15 min prior to NP dosing at varying concentrations. Four hours after NP treatment, cells were washed and analyzed for NP association. Shown are representative NP fluorescence histograms of HM‐1 cells dosed with 30 µg mL^−1^ of PLE‐NPs e), UL‐NPs f), or HA‐NPs g) with or without V9302 compared to untreated cells, and the percentage of NP‐positive cells in PLE‐NP‐treated h), UL‐treated i), or HA‐NP treated j) HM‐1s with or without V9302 across a range of NP concentrations (mean ± s.d.). Statistical comparison in a)–c) was performed via two‐way ANOVA with Tukey's multiple‐comparisons test comparing groups to untreated cells. Data are representative of at least two independent experiments with *n* = 3 technical replicates per group.

Given the potential for polypeptide coatings to interact with amino acid transporters, we next evaluated whether amino acid transport inhibitors could influence LbL‐NP binding. We focused on PLE‐coated LbL‐NPs as our model system based on their unique cell membrane retention property compared to other NPs and promising preclinical utility for therapeutic targeting of ovarian cancer and glioblastoma.^[^ ^7^, ^9^, ^11^, ^13^, ^26^ ^]^ HM‐1 cells were pretreated with various concentrations of the glutamine uptake inhibitors L‐γ‐Glutamyl‐p‐nitroanilide (GPNA) or V9302. We then added fluorescently labeled LbL‐NPs to the cells—comparing UL‐ and PLE‐NPs with NPs layered with HA, polyacrylic acid (PAA), or dextran‐sulfate (DXS) (Figure 2a). We utilized these controls, as HA‐NPs are known to bind CD44, PAA‐NPs have reduced organ‐level accumulation in vivo with minimal cell‐specific interactions, and DXS‐NPs are known to associate with immune cells over cancer cells.^[^ ^44^, ^45^ ^]^ Across these LbL‐NP groups, both glutamine uptake inhibitors enabled a significant decrease in cell association only for PLE‐NPs (Figure 2a–c), which supports the identification of glutamine transporters as binders for PLE‐NPs. Consistent with its ∼100‐fold greater potency in glutamine uptake inhibition relative to GPNA,^[^ ^46^ ^]^ V9302 exhibited a significantly stronger effect on PLE‐NP binding. Further, both glutamine uptake inhibitors were able to demonstrate dose‐dependent blocking of PLE‐NPs, while TFB‐TBOA—a potent aspartate and glutamate uptake inhibitor^[^ ^47^ ^]^—did not affect PLE‐NP binding even at concentrations 1000× higher than its reported IC_50_ (∼10–100 nM) (Figure 2a).

To assess NP association on a single‐cell level, we preincubated HM‐1 cells with a dose of V9302 shown previously to block more than 90% of glutamine uptake (100 µM^[^ ^46^ ^]^) and then dosed with varying concentrations of either UL‐, PLE‐, or HA‐NPs and quantified the percentage of NP‐positive HM‐1 cells. While V9302 showed no impact on association for both UL‐ and HA‐NPs, the association of the PLE‐NPs was significantly negatively impacted (Figure 2d–i). In total, these results support that glutamine transporters play a major role in regulating the binding of PLE‐NPs to cancer cells.

### Availability of SLC1A5 Glutamine Transporter Modulates PLE‐LbL NP Binding to Cancer Cells

Both GPNA and V9302 can block glutamine import by acting on various glutamine transporters known to be overexpressed on cancer cells, including SLC38A2, SLC7A5, and SLC1A5.^[^ ^46^, ^48^ ^]^ Of these, both SLC7A5 and SLC1A5 were identified as associated with PLE‐NP binding in our nanoPRISM analysis. SLC38A2 has limited activity in most cancer cells unless deprived of aminoacids,^[^ ^49^ ^]^ suggesting it might be less likely to drive the PLE‐NP association, which we confirmed using an amino acid transporter inhibitor for SLC38A2, α‐(methylamino)isobutyric acid (MeAIB), which did not affect the PLE‐NP association (Figure S2). While both SLC1A5 and SLC7A5 were considered potential PLE‐NP binders, SLC7A5 a) depends on SLC3A2 complexation for activity, which likely introduces steric hindrance, b) has substantially lower glutamine affinity than SLC1A5, and c) primarily serves for glutamine efflux, limiting its relevance as a candidate for extracellular NP binding.^[^ ^18^, ^50^, ^51^ ^]^ Thus, we focused further investigation on SLC1A5—a glutamine transporter with known overexpression in most cancer types and validated capacity for glutamate binding.^[^ ^52^, ^53^, ^54^, ^55^ ^]^

**Figure 3:**
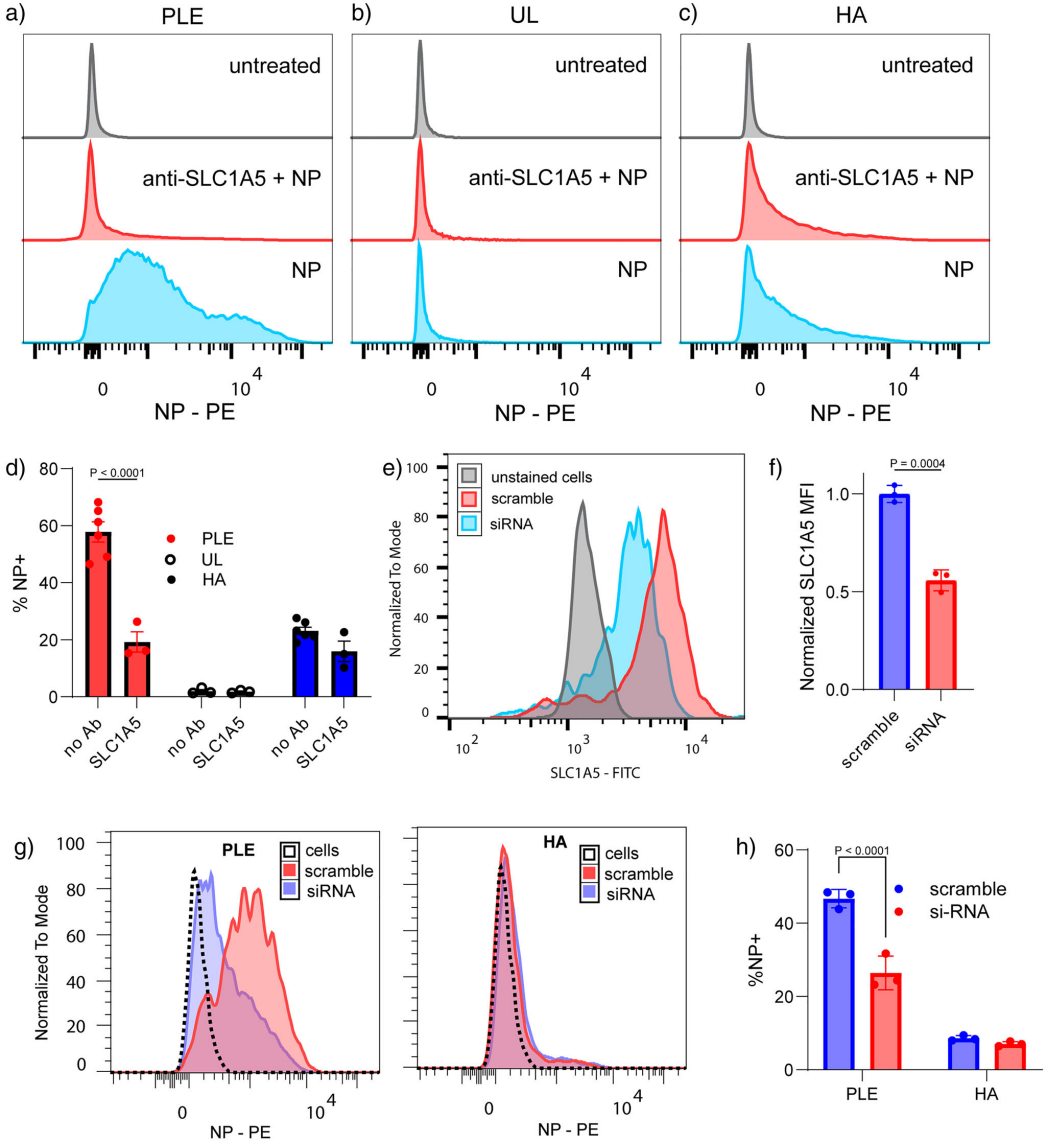
Modulation of SLC1A5 availability regulates PLE‐NP binding. a)–d) HM‐1 cells were treated with antibodies (Abs) against SLC1A5 for 1 h. Fluorescent UL, PLE, or HA NPs (10 µg mL^−1^) were added for 15 min, then cells were washed and analyzed by flow cytometry. Shown are representative histogram plots of NP fluorescence for cells incubated with PLE‐NPs a), UL‐NPs b), or HA‐NPs c) in the presence of anti‐SLC1A5 antibody or control treatments and the percentage of NP + cells for each treatment (mean ± s.e.m., d). e)–h) HM‐1 cells were pre‐treated with either anti‐SLC1A5 siRNA or scrambled siRNA at 200 nM for 96 h before dosing with 10 µg mL^−1^ of NPs for 30 min. After NP incubation, cells were washed with PBS and analyzed with flow cytometry to determine NP association. Shown are representative flow cytometry of anti‐SLC1A5 Ab staining in cells treated with either scramble or anti‐SLC1A5 siRNA e), quantitation of median fluorescence intensity (MFI) of total anti‐SLC1A5 staining (mean ± s.d., f), representative flow cytometry histograms of NP fluorescence of HM‐1 cells with partial SLC1A5 knockdown for PLE‐NP and HA‐NP treatments g), and the percentage of NP positive cells with partial SLC1A5 knockdown treated with PLE‐NP or HA‐NP (mean ± s.d., h). Statistical comparison in d) and h) was performed via two‐way ANOVA with Tukey's multiple‐comparisons test and an unpaired *t*‐test for f). Data are representative of at least two independent experiments with *n* = 3 technical replicates per group.

To further probe the relationship between PLE‐NP binding and SLC1A5 expression, we utilized antibody blocking and gene knockdown experiments to observe the impact on PLE‐NP association. First, to selectively block SLC1A5, we dosed HM‐1 cells with antibodies directed against the transporter before dosing with either PLE‐NP, UL‐NPs, or HA‐NPs. Anti‐SLC1A5 Ab dramatically reduced PLE‐NP association without affecting UL or HA‐NP association (Figure 3a–d), confirming the specificity of PLE‐NP binding to the glutamine transporter. As a cell membrane receptor control, we also evaluated the effect of an anti‐CD44 Ab. Only anti‐SLC1A5 and not anti‐CD44 Abs (Figure S3) could dramatically reduce PLE‐NP association (Figure 3a–d). Next, we performed a transient knockdown of SLC1A5 gene expression via siRNAs to partially reduce total SLC1A5 levels in HM‐1 cells, reaching approximately 50% reduction in SLC1A5 expression compared to cells treated with a scrambled siRNA (Figure 3e,f). SLC1A5 siRNA‐treated HM‐1 cells with reduced SLC1A5 protein expression showed a significant reduction in association with PLE‐NPs, while HA‐NP binding was low and unaffected (Figure 3g,h). Together, these data suggest that PLE‐NPs are capable of directly binding to SLC1A5.

### SLC1A5 Clustering Prolongs Surface Retention of PLE‐NPs

**Figure 4:**
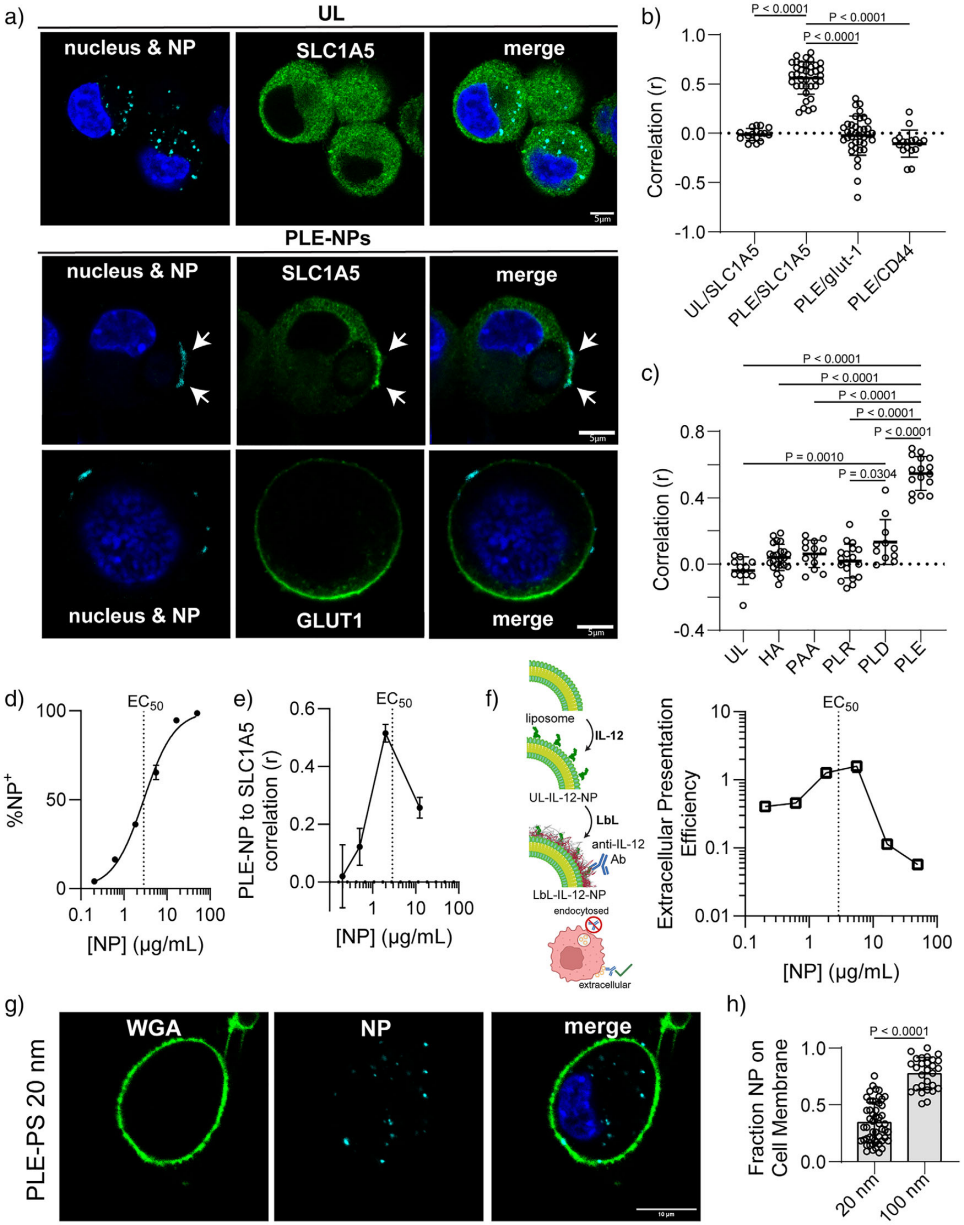
PLE‐NPs colocalize with SLC1A5 transporters on the cell surface. a)–c) HM‐1 cells were dosed with 1.5 µg mL^−1^ of NPs for 2 h, washed with PBS, fixed, permeabilized with saponin, and stained. Shown are representative images of HM‐1 cells treated with UL‐NPs or PLE‐NPs and stained for SLC1A5 or GLUT‐1 transporters a). A correlation analysis between NP signal and cell membrane transporter stain (mean ± s.d., b), and a correlation analysis between varied NP formulations and SLC1A5 staining (mean ± s.d., c) were conducted. Each data point represents correlation across a single cancer cell. d) HM‐1 cells were dosed with varying concentrations of PLE‐IL12 NPs for 4 h. Shown are the percentages of NP‐positive cells for each concentration of NP dosed as determined with flow cytometry. e) The same process as a)–c) was followed with various concentrations of LbL‐NPs tested. Shown is the correlation between the NP signal and the SLC1A5 stain (mean ± s.d.). f) HM‐1 cells were dosed with varying concentrations of PLE‐IL‐12 NPs for 4 h and then stained with anti‐IL‐12 antibody. Shown is the extracellular IL‐12 presentation efficiency (the ratio between extracellular IL‐12 stain to total NP uptake at 24 h compared to the same ratio at 4 h after dosing, mean ± s.d.). g), h) HM‐1 cells were dosed with 1 µg mL^−1^ of NPs for 4 h, washed with PBS, fixed, and stained with Hoechst 33342 and WGA for visualization on a confocal microscope. Shown are representative HM‐1 cells dosed with 20 nm PLE‐PS particles g) and the quantification of the fraction of NP pixels colocalized with cell membrane pixels (mean ± s.d., h). Statistical comparison in b) and c) was performed via two‐way ANOVA with Tukey's multiple‐comparisons test and an unpaired *t*‐test for h). Data are representative of at least two independent experiments. Scale bars in a) and g) represent 5 and 10 µm, respectively.

Having discovered a binding target of PLE‐NPs, we theorized that the SLC1A5 interaction might also contribute to the high cell surface retention of PLE‐NPs observed in previous work, where we have successfully leveraged this property to deliver interleukin‐12 (IL‐12) to the tumor extracellular microenvironment.^[^ ^7^, ^13^, ^26^ ^]^ To determine if SLC1A5 is associated with this surface retention, we utilized confocal microscopy to image HM‐1 cells incubated with fluorescently‐labeled IL‐12‐NPs with or without a PLE coating. We then stained to visualize the cellular localization of SLC1A5, revealing that UL‐NPs were rapidly endocytosed with no signs of interaction with SLC1A5 (Figures 4a and S4a). By contrast, PLE‐NPs formed clusters on the cell surface of HM‐1 cells that colocalized with accumulated SLC1A5 (Figures 4a and S4a, white arrows). The correlation of NPs with cell surface receptors was specific to SLC1A5, as neither CD44 nor GLUT‐1 colocalized with PLE‐NPs (Figures 4a,b and S4b,c). We confirmed this correlation of NPs with SLC1A5 was specific to PLE NPs, as other LbL‐NP coatings—including a PLR monolayer or bilayers with PLR followed by HA or PAA—did not show a significant correlation even when the NPs were found on the surface of cancer cells (Figures 4c and S4d). PLD NPs had a low but statistically significant level of correlation with SLC1A5 when on the cell surface (Figures 4c and S4d), suggesting that a polyaspartate coating may also be engaging this amino acid transporter.

While SLC1A5 itself has a low internalization rate (half‐life 20–60 h^[^ ^56^, ^57^ ^]^) that could aid in the surface retention of PLE‐NPs, we hypothesized that the cell surface clustering of NPs bound to SLC1A5 could contribute to their high cell surface retention property, similar to certain galectin lattice structures.^[^ ^58^ ^]^ To evaluate the effect of clustering, we dosed increasing concentrations of NPs to HM‐1 cells and either imaged cells via confocal microscopy or quantified NP association via flow cytometry. Near the NP EC_50_ (the NP concentration leading to half‐maximal binding of particles), we could observe a clear increase in the correlation between PLE‐NPs and SLC1A5 signal (Figure 4d,e), suggesting an increase in cluster size or clustering efficiency. However, NP doses above the EC_50_ reduced SLC1A5 clustering without affecting the total fraction of initial NP binding to the cell surface (Figures 4d,e and S5a,b), likely due to the saturation of SLC1A5 receptors on the cell surface (i.e., preventing one NP from binding multiple receptors). Indeed, staining for SLC1A5 showed a lack of PLE‐SLC1A5 foci formation at high doses of PLE‐NPs (Figure S5c).

To determine the relationship between transporter clustering and NP internalization, we leveraged the presence of a therapeutic cargo on the NPs to probe its delivery to the cell surface or intracellularly. For this experiment, IL‐12 was conjugated to fluorescent liposome surfaces, followed by LbL layering of PLR and PLE (Figure 4f). We have previously shown that IL‐12 on LbL‐coated particle surfaces is accessible to staining with an anti‐IL‐12 monoclonal antibody. IL‐12 PLE‐NPs were incubated with HM‐1 cells followed by staining with anti‐IL‐12, and the extracellular IL‐12 staining signal was compared to the total NP signal from flow cytometry at 4 and 24 h after dosing with various concentrations of NPs. Below the EC_50_, NPs were effectively retained on the cell surfaces, with ∼50% of the IL‐12 signal remaining extracellular at 24 h, consistent with a ∼20 h half‐life^[^ ^57^ ^]^ of SLC1A5 (Figure 4f). Strikingly, there was little to no NP uptake near the EC_50_ as the ratio of external IL‐12 signal to total NP signal remained constant (value ∼1). On the other hand, NPs incubated with HM‐1 cells at concentrations well above the EC_50_ showed a strong decline in the IL‐12:NP signal ratio, indicating internalization and suggesting that a lack of receptor clustering at high NP doses may facilitate particle internalization.

While surface retention of PLE‐NPs is desirable for the delivery of drugs targeting the extracellular space, certain drug delivery applications may benefit from rapid NP internalization. Consistent with the clustering‐induced surface retention of the PLE‐NPs mechanism presented here, we have previously shown that the addition of a co‐polymer of PLE with polyethylene glycol (PEG) to the LbL film prevents surface retention of PLE‐NPs on cancer cells.^[^ ^9^ ^]^ PEG may act to sterically inhibit cluster formation at the cell surface. We theorized that the faster internalization kinetics of small NPs may also avoid cluster assembly.^[^ ^59^ ^]^ Moreover, as SLC1A5 has been previously shown to colocalize with caveolin‐1, small PLE‐NPs may readily fit into caveolar pits, which could avoid cluster formation and allow for rapid internalization.^[^ ^60^, ^61^ ^]^ We thus coated carboxylated PS NPs of either 20 or 100 nm in diameter with a PLE LbL film. As reported previously,^[^ ^7^ ^]^ 100 nm PLE‐NPs accumulated on the cell surface (Figure S6). However, when we dosed HM‐1 cells with 20 nm PLE‐NPs, we could readily observe internalized PLE‐NPs within 4 h after dosing (Figure 4g,h). In all, these results suggest that inducing the clustering of SLC1A5 transporters confers surface retention for PLE‐NPs.

### Molecular Modeling Predicts Polypeptide Interactions with SLC1A5 and Unique PLD‐SLC1A3 Interactions

Although colocalization of PLE‐NPs with SLC1A5 was observed, the interaction between NPs and cells in biological fluids remains highly complex due to the potential formation of a protein corona.^[^ ^62^ ^]^ Previous studies demonstrated that the PLE coating substantially reduces protein corona formation, which may allow for more direct interactions between the NP surface and cell membrane components such as SLC1A5.^[^ ^63^ ^]^ Nonetheless, exposure to biological fluids may still influence NP uptake and cellular interactions. Moreover, SLC1A5 and other amino acid transporters evolved to transport individual amino acid monomers and not polypeptides. Thus, to further validate and potentially gain insights into how glutamate polymers such as the one in the LbL coating might interact with SLC1A5, we turned to computation modeling with AlphaFold 3.^[^ ^64^ ^]^

We probed the human sequences for SLC1A5 and SLC38A2 as positive and negative controls for PLE‐NP interactions given the results from small molecule inhibition. To further evaluate the potential interaction with additional glutamine transporters, we also evaluated interaction with SLC7A5. Transporters were modeled interacting with small (*n* = 4) oligomers of PLE, poly‐L‐glutamine (PLQ), or a control polypeptide with low SLC1A5 interaction—poly‐L‐phenylalanine (PLF) (Figure S7a).^[^ ^65^ ^]^ We also included PLD in the structure prediction modeling to gain more insights into the potential differences between PLE and PLD. Previously, we demonstrated that while PLE‐NPs remain on the cell surface for extended durations, PLD‐NPs are gradually internalized via caveolin‐mediated endocytosis following their binding to cancer cell surfaces, which may be due to differences in their targets.^[^ ^7^ ^]^ Given the ability of AlphaFold 3 to include ions and the requirement of sodium by these transporters, they were included in the model.^[^ ^66^, ^67^ ^]^ Model prediction was performed at least twice, and the chain‐pair interface predicted template modeling (ipTM) score was extracted from the top‐ranked predictions. The ipTM score is used to rank a specific interface between two chains, with values above 0.8 representing confident high‐quality predictions, values above 0.6 suggesting potential interactions, and values below 0.3 as non‐interacting.^[^ ^64^, ^68^ ^]^ Notably, these simulation do not capture the spatial arrangement of the polymers on the NP surface nor the direct mechanism of interactions of the amino acid transporters with large polymers; rather, the resulting data are intended to demonstrate the potential for binding to occur.

**Figure 5:**
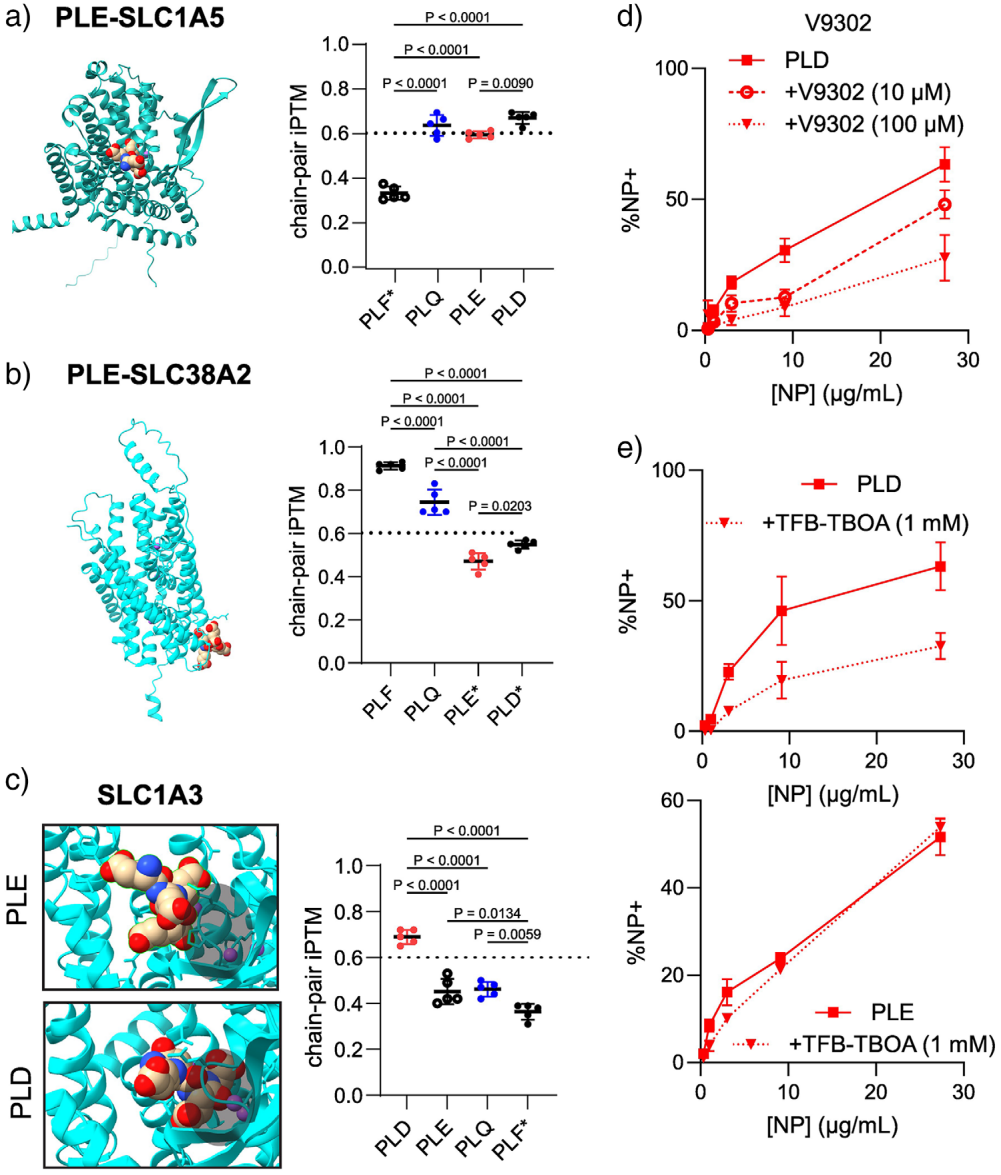
The AlphaFold model predicts polymer binding to amino acid transporters. a) Representative AlphaFold 3 model structure of SLC1A5 and PLE and the calculated chain‐pair ipTM scores between SLC1A5 with PLF, PLQ, PLE, and PLD (mean ± s.d.). b) Representative AlphaFold 3 model structure of SLC38A2 and PLE and the calculated chain‐pair ipTM scores between SLC38A2 with PLF, PLQ, PLE, and PLD (mean ± s.d.). c) Representative AlphaFold 3 model structure of SLC1A3 and PLE and SLC1A3 with PLD focused on the binding pocket indicated by the dark shading. AlphaFold 3 calculated chain‐pair ipTM scores between SLC1A3 with PLD, PLE, PLQ, and PLF (mean ± s.d.). d), e) HM‐1 cells were treated with either V9302 or TFB‐TBOA for 15 min before NP dosing at varying concentrations. Two hours after NP treatment, cells were washed with PBS and analyzed with flow cytometry to measure NP association. Shown are the percentage of NP‐positive cells in PLD‐treated HM‐1s with V9302 (mean ± s.d., d) and the percentage of NP‐positive cells in PLE‐NP or PLD‐NP treated (mean ± s.d., e) HM‐1s with 1 mM of TFB‐TBOA. (* in AlphaFold 3 iPTM scores indicates polymer not in known binding pocket of transporter). Statistical comparison in a), b), and c) was performed via two‐way ANOVA with Tukey's multiple‐comparisons test.

In the evaluation of SLC1A5, PLE and PLQ were found inside the binding pocket of the protein in its conformation facing the extracellular environment (i.e., outward‐facing) with ipTM scores indicative of binding (∼0.6, Figure 5a). Consistent with the enrichment for glutamine transporters in its gene sets (Figure 1e) and the partial colocalization of PLD with SLC1A5 observed by confocal microscopy (Figure 4c), PLD also showed favorable interaction with SLC1A5, suggesting that PLD‐NPs and PLE‐NPs may both interact with similar amino acid transporters that are overexpressed in cancer cells. On the other hand, PLF had significantly reduced ipTM scores and was found outside of any known pocket (Figure S7b). Performing the same analysis on SLC38A2 with PLE, PLD, and PLQ showed that only PLQ interacted favorably (ipTM > 0.6) with the binding pocket, whereas PLE and PLD had significantly lower ipTM scores and did not bind to a known pocket of SLC38A2 (Figures 5b and S7c). PLF, on the other hand, yielded a high ipTM score with SLC38A2, potentially due to the minor transport capacity of phenylalanine by this transporter.^[^ ^49^ ^]^ Moreover, only PLQ favorably interacted with SLC7A5, indicating a specificity of PLE and PLD to SLC1A5 (Figure S7d).

Even though specialized transporters for glutamate and aspartate exist, PLE‐NPs were not found to interact with these, as TFB‐TBOA did not inhibit PLE‐NP association (Figure 2b). To better understand this observation, we simulated the interaction of PLE and PLD with SLC1A3, an anionic amino acid transporter that has been implicated as a major contributor to cancer progression and is overexpressed in many solid tumors.^[^ ^69^, ^70^, ^71^, ^72^ ^]^ AlphaFold predictions showed that PLE could bind near the pocket but did not fit inside given the size of PLE's side chain (Figure 5c). On the other hand, PLD was predicted to bind inside the pocket. Indeed, quantification of chain‐pair ipTM scores showed that PLD had favorable binding to SLC1A3, compared to PLE's significantly lower score, which was similar to PLQ (Figure 5c). PLF had the lowest ipTM score and was found outside of any known binding pocket.

Based on the predicted interactions of PLD with both SLC1A5 and SLC1A3, we next sought to validate these observations experimentally. We first evaluated if the glutamine transport inhibitor V9302, found to block PLE‐NP binding, could also block PLD‐NP association. V9302 showed a dose‐dependent inhibition of PLD‐NP binding to HM‐1 cells (Figure 5d), indicating PLD‐NPs also bound to SLC1A5. We next dosed HM‐1 cells with the anionic amino acid transport inhibitor—TFB‐TBOA—to evaluate its effects on PLD‐NPs and PLE‐NP association at the single‐cell level via flow cytometry. Consistent with the structure modeling predictions, TFB‐TBOA partially blocked PLD‐NP but not PLE‐NP association across NP doses (Figure 5e). Importantly, this ability of PLD NPs to bind to anionic amino acid transporters such as SLC1A3 explains the increased rate of PLD over PLE endocytosis since transporters of anionic amino acids have a much shorter surface half‐life (<1 h^[^ ^73^, ^74^ ^]^) compared to SLC1A5 (20–60 h^[^ ^56^, ^57^ ^]^). Further, PLD‐NP binding to SLC1A3 is consistent with the previously shown caveolin‐mediated NP trafficking, as SLC1A3 uptake is mediated through caveolin.^[^ ^73^, ^75^ ^]^ Thus, unlike PLE, PLD coating maintains binding to anionic amino acid transporters, allowing for accelerated internalization rates.

### Expression of Amino Acid Transporters Correlates with PLE and PLD NP Association Across Various Human Cancer Cell Lines

SLC1A5 and SLC1A3 are both overexpressed in many human cancers, with expression correlating with poor prognosis.^[^ ^41^, ^55^, ^76^, ^77^, ^78^, ^79^ ^]^ Given the trends observed above, we theorized that the expression of these receptors may be predictive of PLE‐ and PLD‐NP binding and could be leveraged to find optimal cancers to target with these platforms. We previously screened a library of LbL‐NPs, including PLE‐ and PLD‐NPs, on various human ovarian cancer cell lines and primary murine non‐cancerous tissues.^[^ ^7^ ^]^ In this study, we screened fluorescently labeled carboxylated PS NPs coated with either no outer layer or one of nine distinct outer layer chemistries. We then quantified cell‐type‐specific preferences for each outer layer using Z‐score analysis.^[^ ^7^ ^]^

**Figure 6:**
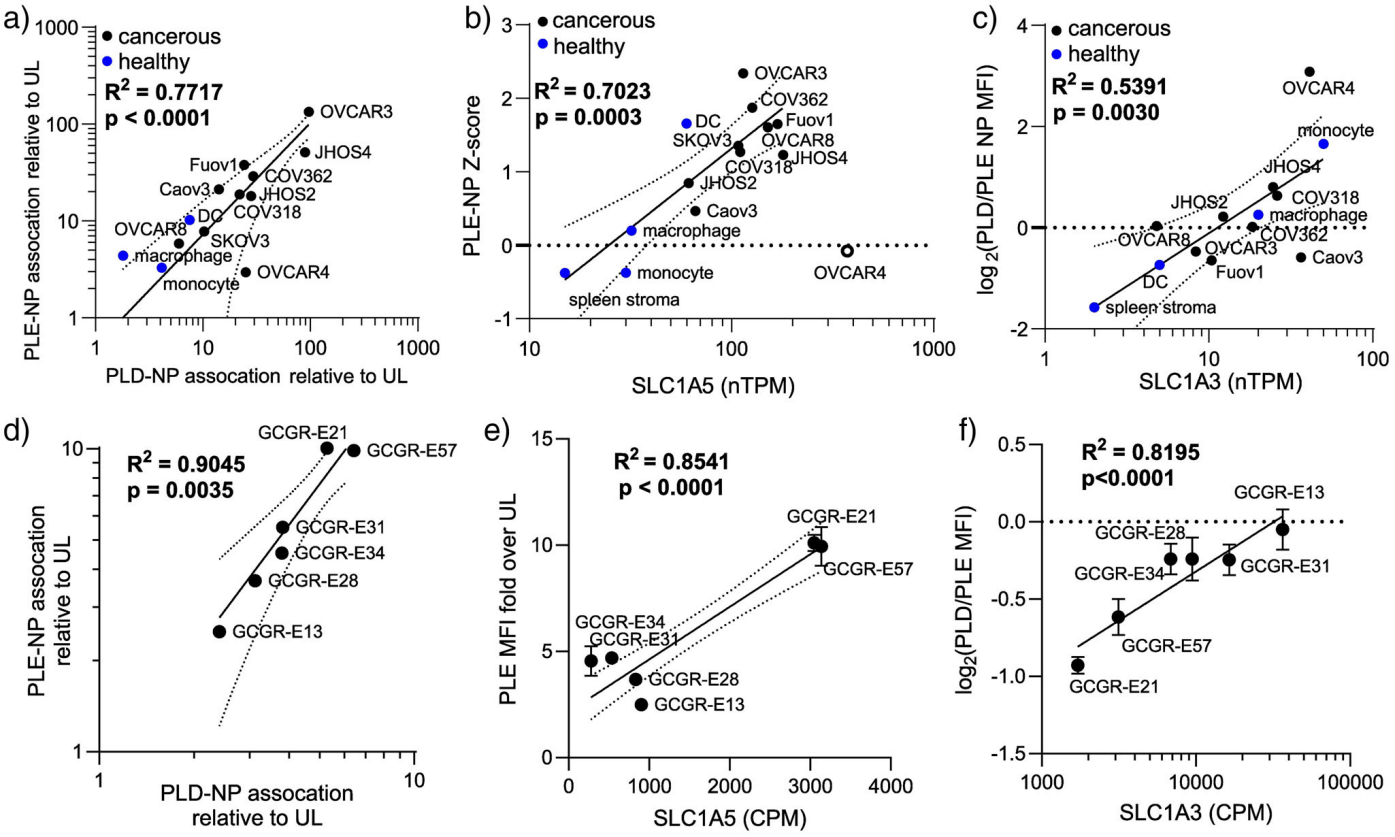
PLE‐NP and PLD‐NP association correlates with SLC1A5 and SLC1A3 expression. a)–c) Analysis of NP association with ovarian cancer cell lines and primary healthy cells with RNA expression of human cell lines derived from Protein Atlas. Shown are log–log plots of fold increase in PLE‐ and PLD‐coated LbL‐NPs relative to UL NPs in a library of ovarian cancer cell lines and primary healthy cells (mean, a), PLE‐NP Z‐scores from NP screen against the same cells as a function of SLC1A5 RNA expression (mean, b), and the ratio of NP MFI of PLD‐NP‐treated cells to the MFI of PLE‐treated cells as a function of SLC1A3 expression (mean, c). d)–f) Same analysis as a)–c) but with glioblastoma cell lines (mean ± s.d.). Dashed lines represent the 95% confidence interval of the curve fit. R^2^ values were derived from linear fits on plots with axes as shown, and *p*‐values were derived from the non‐zero slope test.

We first evaluated the correlation between PLE and PLD NPs across the cell lines, as the shared binding toward SLC1A5 should yield similar associations across cells. Indeed, there was a strong correlation between the increase in NP association of PLE and PLD over UL NPs in 14 human ovarian cancer cell lines and primary murine cells (Figure 6a). To determine if genetic markers could predict the specificity of interaction with cells, we correlated the expression levels of SLC1A5^[^ ^80^ ^]^ to their preference toward PLE‐NPs over other NPs (PLE‐NP Z‐score). Except for one cell line (OVCAR4), we observed a strong relationship between PLE‐NP preference and its SLC1A5 expression level (Figure 6b). OVCAR4 may be an outlier due to its high expression of hypoxia‐related genes (Figure S8), which induce an intracellularly restricted isoform of SLC1A5.^[^ ^81^ ^]^

As expected, a similar correlation was observed between the SLC1A5 and PLD‐NP association (Figure S9a). However, no correlation was observed for SLC7A5 or SLC38A2, confirming the specificity of these NPs to SLC1A5 (Figure S9b–e). We next decided to explore if the expression of SLC1A3 would be predictive of the difference in binding of PLD‐NPs to cells compared to PLE‐NPs. We correlated the log‐fold increase in PLD‐NPs binding over PLE‐NPs in these cells to their SLC1A3 expression and found a clear correlation (Figure 6c). Notably, hypoxia‐related genes induce SLC1A3 expression and activity such that OVCAR4 showed the highest preference toward PLD over PLE.^[^ ^82^ ^]^

In addition to ovarian cancer cells, we previously showed that PLE‐NPs are highly selective toward glioblastoma in vitro and in vivo.^[^ ^9^, ^11^ ^]^ Consistent with these experimental observations, brain tumors are one of the cancers with the highest fold change in SLC1A5 gene expression compared to healthy tissue.^[^ ^77^ ^]^ Thus, to confirm the role of SLC1A5 expression levels with PLE‐ and PLD‐NP binding, we screened their association to glioblastoma cell lines. We incubated six glioblastoma cell lines with UL‐, PLE‐, or PLD‐NPs and quantified total NP fluorescence via flow cytometry. Similar to the ovarian cancer cell analysis, PLE‐ and PLD‐NP delivery were found to be highly correlated (Figure 6d). Moreover, there was a clear relationship between the expression levels of SLC1A5 in the tested glioblastoma cell lines and the enhancement in NP association due to PLE (Figure 6e) or PLD coating (Figure S9f). Last, the ratio of association with PLD‐ over PLE‐NPs also correlated with SLC1A3 expression in these glioblastoma lines (Figure 6f). In all, these analyses suggest that SLC1A5 expression correlates with both PLE‐NP and PLD‐NP delivery, while SLC1A3 expression may drive differences in cell association for PLE‐ and PLD‐NPs.

## Conclusion

Targeting overexpressed surface markers on cancer cells to achieve specific therapeutic delivery to tumors is a major goal of drug delivery carriers. Negatively charged polypeptide LbL coatings, including PLE, have empirically demonstrated in vivo targeting of cancerous tissues in multiple murine models and enabled control over cellular trafficking, but their binding targets on the cancer cell surface have remained unknown.^[^ ^7^, ^10^, ^13^, ^37^ ^]^ Here, we report that the high avidity presentation of anionic polypeptide coatings on LbL NPs confers targeting to amino acid transporters overexpressed in cancerous tissues. LbL coating was optimal for the proper amino acid sidechain presentation to cancer cells, whereas simply grafting polypeptide chains to NPs inhibited cell association.

To determine the surface biomarkers that act as targets for these systems, we mined prior large NP screening experiments^[^ ^27^ ^]^ and identified glutamine amino acid transporters within gene sets predictive of polypeptide LbL‐NP association. Through small molecule inhibition, antibody blocking, and gene knockdown, we identified SLC1A5—a glutamine transporter overexpressed in many cancer types—as the major target of PLE‐NPs. Further, we observed clear colocalization of PLE‐NPs with SLC1A5 on the cell surface and discovered that the induced clustering of SLC1A5 enables the surface anchoring properties associated with PLE‐NPs. Based on this observation, we show that smaller PLE‐NPs are more readily internalized, which is consistent with a reduced SLC1A5 clustering at smaller particle sizes. This framework also provides an explanation for our prior observation that PEG‐PLE copolymer LbL‐NPs display higher internalization than PLE LbL‐NPs, as PEG shielding likely prevents PLE‐mediated SLC1A5 clustering.^[^ ^9^ ^]^ Combining small molecule inhibition with artificial intelligence protein structure prediction, we also show that unlike PLE‐NPs, PLD‐NPs interact with both SLC1A5 and SLC1A3. This dual targeting of PLD explains the difference in internalization compared to PLE NPs, as SLC1A3 has higher internalization rates that proceed through caveolin‐driven mechanisms as compared to SLC1A5.^[^ ^56^, ^57^, ^73^, ^74^, ^75^ ^]^ Moreover, we confirm these binding interactions by analyzing LbL‐NP screens with ovarian cancer and glioblastoma cell lines, which demonstrate a clear correlation between SLC1A5 expression with PLE‐ and PLD‐NP association and that the preference for PLD‐NPs over PLE‐NPs was related to the expression of SLC1A3.

Given the increased expression of amino acid transporters in cancer cells, they have been prior targets for drug delivery. Prior work developed glutamine‐grafted polymers to target amino acid transporters such as SLC1A5.^[^ ^83^, ^84^ ^]^ However, as we show here, through the LbL coating, we can achieve dramatically higher affinity given its unique ability to increase surface avidity and surface presentation while enabling the design of NPs that can be retained on the cancer cell surface for extended periods. Interestingly, glutamate has been used as a control compared to glutamine with little discussion on the potential glutamate‐SLC1A5 interaction, which we find drives PLE‐NP binding.^[^ ^83^, ^84^ ^]^ Glutamate conjugation via the side chain carboxylic acid has also been employed, but such an approach led to no interaction with SLC1A5 and unclear binding partners, likely due to the nature of the presentation of the amino and carboxyl groups present on all amino acids.^[^ ^85^, ^86^ ^]^ Grafting of glutamine residues onto branched polyethylenimine (PEI) for polyplexes has also been used;^[^ ^87^ ^]^ however, glutamine residues are more likely to interact with other glutamine transporters such as SLC38A2 and SLC7A5. Moreover, unlike LbL‐NPs, these particles lack control over internalization rates.

Beyond avidity, the high affinity of PLE and PLD toward SLC1A5 may originate from the induced protonation of their anionic residues. Polyelectrolytes tend to shift the monomer pKa due to unfavorable charge repulsion of residues in proximity. Indeed, PLE and PLD have an apparent pKa of ∼6.0^[^ ^88^ ^]^ even though the side chains of monomeric glutamate and aspartate residues have pKa of 4.3 and 3.9, respectively.^[^ ^89^ ^]^ Interestingly, the transport and binding of glutamate to SLC1A5 is pH dependent, with greater transport at pH 6.0 than pH 7.0.^[^ ^52^, ^53^, ^54^ ^]^ In addition to potentially affecting charges in the binding pocket, direct proton transport is required in this system, with protonated glutamate (i.e., glutamic acid) estimated to have dramatically higher affinity toward SLC1A5 compared to its charged counterpart.^[^ ^53^, ^54^ ^]^ However, more work is required to validate whether the protonated glutamate is transported or if proton transport occurs via a separate path.^[^ ^52^ ^]^ Unfortunately, our protein structure modeling predictions do not account for local pKa factors, which likely lead to underestimated interactions between PLE and PLD with SLC1A5. Further, the simulation used in this work does not capture several factors that influence the spatial conformation of PLE and PLD, including polymer arrangement within the LbL surface film, protein corona formation, and NP‐related properties such as size and stiffness, all of which can affect interactions with the cell surface.^[^ ^33^, ^62^, ^63^ ^]^ To overcome this, future studies employing physics‐based protein interaction models that more accurately account for the local pKa environment within the binding pockets of amino acid transporters and the behavior of longer charged polypeptide chains may help clarify potential binding modes and affinities of these polymers. Incorporating the NP surface into such models could further provide insights into how polymer conformation within LbL films contributes to amino acid transporter binding.

Future work may aim to better understand the role of anionic amino acid transporters in mediating PLD‐NP internalization. For clinical translation, it will be critical to evaluate how the surface presentation of these transporters and their expression levels on patient samples correlate with NP binding. Taken together, the data presented here provides the first demonstration that high avidity presentation of anionic polypeptides from LbL‐NPs enables targeting to overexpressed amino acid transporters. These insights not only guide future LbL‐NPs applications but also now enable future rational design of next‐generation polymeric coatings. Notably, the nature of these binding interactions provides insights for future clinical NP applications across multiple cancer types.

## Supporting Information

The data that support the findings of this study are available in the supplementary material of this article. The authors have cited additional references within the Supporting Information.^[^ ^90^, ^91^, ^92^, ^93^, ^94^ ^]^

## Conflict of Interests

I.S.P., P.T.H. and D.J.I. are inventors on patents filed by the Massachusetts Institute of Technology relating to LbL NP therapeutics. The other authors declare no competing interests.

## Supporting information



Supporting Information

## Data Availability

The data that support the findings of this study are available from the corresponding author upon reasonable request.
