# Supplementary material for: Surface Avidity of Anionic Polypeptide Coatings on Layer‐by‐Layer Nanoparticles Target Cancer‐Associated Amino Acid Transporters
Source: Angew Chem Int Ed Engl. 2025 Dec 22;65(8):e19203. doi: 10.1002/anie.202519203 (PMC12910144; doi:10.1002/anie.202519203)
Supplement: Supplementary file 1 — Supporting Information [file ANIE-65-e19203-s001.docx]

**Supplementary Information**

**Surface Avidity of Anionic Polypeptide Coatings on Layer-by-Layer Nanoparticles Target Cancer-Associated Amino Acid Transporters**

*Ivan S. Pires^[a,b]^, Margaret M. Billingsley^[a]^, Ezra Gordon^[b]^, Andrew J. Pickering^[a,b]^, Eva Cai^[a,c]^, Gonzalo J. Esparza^[b]^, Mae L. Pryor^[a,e]^, Alexander D. Stoneman^[a,b]^, , Aidan Kindopp^[a,b]^, Darrell J. Irvine*^[a,d,e,f,g]^ Paula T. Hammond*^[a,b]^*

[a] Dr. I. S. Pires, Dr. M. M. Billingsley, Dr. A. J. Pickering, E. Cai, M. L. Pryor, A. D. Stoneman, Prof. D. J. Irvine, Prof. P. T. Hammond

Department: Koch Institute for Integrative Cancer Research

Institution: Massachusetts Institute of Technology

Address: 500 Main Street, Cambridge, Massachusetts 02139, United States

Emails: djirvine@mit.edu, hammond@mit.edu

[b] Dr. I. S. Pires, E. Gordon, Dr. A. J. Pickering, G. J. Esparza, A. D. Stoneman, A. Kindopp, Prof. P. T. Hammond

Department: Department of Chemical Engineering

Institution: Massachusetts Institute of Technology

Address: 21 Ames Street, Cambridge, Massachusetts 02139, United States

[c] E. Cai

Department: Harvard-MIT Health Sciences and Technology

Institution: Massachusetts Institute of Technology

Address: 77 Massachusetts Ave, Cambridge, MA 02139, United States

[d] Prof. D. J. Irvine

Department: Department of Biological Engineering

Institution: Massachusetts Institute of Technology

Address: 25 Ames Street, Cambridge, Massachusetts 02139, United States

[e] M. L. Pryor, Prof. D. J. Irvine

Department: Department of Materials Science and Engineering

Institution: Massachusetts Institute of Technology

Address: 182 Memorial Dr, Cambridge, MA 02139, United States

[f] Prof. D. J. Irvine

Institution: Ragon Institute of MGH, MIT and Harvard University

Address: 600 Main St, Cambridge, MA 02139, United States

[g] Prof. D. J. Irvine

Institution: Howard Hughes Medical Institute

Address: 4000 Jones Bridge Rd, Chevy Chase, MD 20815, United States

**Table of Contents**

Materials S3

Supplementary Figures S10

**Methods**

*Materials*

1,2-distearoyl-sn-glycero-3-phosphocholine (DSPC), 1,2-dioleoyl-sn-glycero-3-phosphoethanolamine-N-[4-(p-maleimidophenyl)butyramide] (sodium salt) (18:1 MPB-PE), 1-palmitoyl-2-oleoyl-sn-glycero-3-phospho-(1’-rac-glycerol) (sodium salt) (POPG), 1,2-dioleoyl-sn-glycero-3-phosphoethanolamine-N-dibenzocyclooctyl (DOPE-DBCO), 1,2-dioleoyl-3-trimethylammonium-propane (chloride salt) (DOTAP), and cholesterol were purchased from Avanti Polar Lipids. Poly-L-arginine (PLR) with a molecular weight (MW) of 9.6 kDa and poly-L-glutamic acid (PLE) with a MWs of 15 kDa (PLE_100_) or 120 kDa (PLE_800_) were purchased from Alamanda Polymers. BDP TMR azide (Lumiprobe) and BDP 630/650 azide (Lumiprobe) were conjugated to DOPE-DBCO in chloroform to generate fluorescently labeled lipids. Successful conjugation was validated via thin-layer chromatography which indicated <1% free dye. Fluorescently-labeled carboxylated polystyrene particles were purchased from ThermoFisher. V9302, α-(Methylamino)isobutyric acid (MeAIB) and TFB-TBOA, were purchased from MedChemExpress and L-γ-Glutamyl-p-nitroanilide (GPNA) was purchased from Sigma. Anti-CD44 antibody (IM7, functional grade), and anti-GLUT-1 antibody (SA0377) were purchased from Invitrogen. For anti-SLC1A5, two clones directed at extracellular epitopes were purchased - AB_2806719 from Invitrogen and AB_2878679 from Proteintech. An intracellular epitope directed anti-SLC1A5 (AB_2756720) was purchased from Alomone Labs. Anti-mouse IL-12 (clone C15.6) was purchased from Biolegend. Deionized water of the ultrapure grade was obtained through a Milli-Q water system (EMD Millipore).

*Recombinant single-chain IL-12 production*

Single-chain IL-12 sequence^[1]^ was synthesized as a genomic block (Integrated DNA Technologies) and cloned into gWIZ expression vector (Genlantis). Plasmids were transiently transfected into Expi293 cells (ThermoFisher Scientific). After 5 days, cell culture supernatants were collected and protein was purified in an ÄKTA pure chromatography system using HiTrap HP Niquel sepharose affinity column, followed by size exclusion using Superdex 200 Increase 10/300 GL column (GE Healthcare Life Sciences). Endotoxin levels in purified protein was measured using Endosafe Nexgen-PTS system (Charles River) and assured to be <5 EU/mg protein.

*Liposome synthesis*

Lipid stocks were stored at -20 °C in amber vials in chloroform. A lipid solution was prepared by mixing DSPC (25 mg/mL), cholesterol (25 mg/mL), and POPG (25 mg/mL) at a 70:24:6 mole % ratio and then forming a thin film using a rotary evaporator (Buchi). Lipid films were allowed to further dry overnight in a desiccator, then were hydrated at 0.5-1 mg/mL using deionized water and sonicated for 3-5 min at 65 °C then extruded (Avestin Liposofast LF-50) at 65 °C once through a 100 nm membrane (Cytiva Nuclepore) then 3 times through 50 nm membranes (Cytiva Nuclepore). Extruded liposomes were cooled in an ice bath. For fluorescence labeling of liposomes, 0.2 mol% of DSPC content was replaced by either DOPE-TMR or DOPE-630/650. After extrusion, lipid concentration was determined based on the fluorescent signal from pre-extruded lipid sample of known concentration. Cationic liposomes were made via same method but replacing POPG with DOTAP.

*IL-12 Liposome synthesis*

For covalent linkage of scIL-12 to liposomes, 5 mol% of DSPC was replaced with 5% of 18:1 MPB-PE (5 mg/L) in the lipid solution prior to lipid film formation and dried in the same method as standard liposomes. For lipid film hydration, the solution pH of MPB-PE liposomes was adjusted to pH 5 with hydrochloric acid to prevent maleimide hydrolysis. Following hydration, liposomes at 0.33 mg/mL were adjusted to pH 7.0 with 10 mM HEPES followed by the addition of scIL-12 containing a terminal cysteine residue at a molar ratio of 25:1 of MPB-PE lipid to protein for at least 12 hrs at 4 °C in a rotating mixer. Any remaining maleimides were quenched with a 100-fold molar excess of L-cysteine (Sigma) for 1.5 hrs on ice. Unlayered IL-12 liposomes were then purified via tangential flow filtration using a 100 kDa (mPES, Repligen) hollow fiber membrane for 7 diafiltration volumes.

For fluorescence labeling of liposomes, 0.2 mol% of DSPC content was replaced by either DOPE-TMR or DOPE-630/650. IL-12 concentrations were measured via enzyme-linked immunoassay (ELISA) (Peprotech) and lipid content was determined based on the fluorescence of the pre-extruded lipid sample of known concentration.

*Layer-by-layer (LbL) film deposition onto nanoparticles*

Assembly of polyelectrolyte layers was performed by adding unlayered particles to a diH_2_O solution with 0.3-0.4 weight equivalents (wt.eq.) of PLR relative to lipid in a glass vial under sonication and incubating on ice for at least 30 min. Excess PLR polymer was purified by tangential flow filtration (TFF) through a 100 kDa mPES membrane (Repligen) pre-treated with a 10 mg/mL solution of free PLR. For the terminal PLE layer, purified particles coated with PLR were added to a diH_2_O solution with PLE in a glass vial under sonication at 1 wt.eq. of polymer to lipid. LbL particles were then purified by TFF on a separate 100 kDa mPES membrane (Repligen) to remove any excess PLE.

For high throughput assembly onto carboxylated polystyrene particles or varied outer-layer chemistries of IL-12 liposomes, LbL assembly was performed using a microfluidics-based method.^[2]^ Briefly, polymer wt.eq. were titrated to core particles based on the amount of polymer required for the onset of the plateau point of zeta potential. Then the particle solution and titrated amount of polymer were mixed using a microfluidics cartridge at equal flow rates of 10 mL/min per channel (Precision Nanosystems).

*Characterization of particle preparations*

Dynamic light scattering (DLS) and zeta potential measurements were made on a Zetasizer Pro (Malvern) with NPs diluted in deionized water at a concentration of approximately 0.1 mg/ml for 3 runs per sample.

*Grafted PLE-liposome assembly*

To generate liposomes with grafted PLE polymers, PLE_100_ with a C-terminal azide (PLE-N3) was purchased from Alamanda Polymers. A lipid solution in chloroform with 69.8 mol% DSPC, 30 mol% cholesterol, 0.2 mol% DOPE-630/650 was allowed to dry overnight in a desiccator then suspended at 10 mg/mL in 10% MEGA-10. A separate solution with 10 mg/mL of DOPE-DBCO in 10% MEGA-10 was made and mixed with 2 molar excess of PLE-N3 and allowed to react overnight at 25 °C to generate DOPE-PLE. The two samples were mixed to generate a lipid solution containing 0.5 mol% of the DOPE-PLE and then liposome assembly was induced by diluting the sample with phosphate-buffered saline (10 mM, pH 7.4) to 0.01% MEGA-10. Samples were concentrated and purified from detergent via TFF in a 100 kDa mPES membrane.

*Fluorescent labeling of PLE polymers*

PLE_100_ or PLE_800_ at 10 mg/mL was labeled by reacting with 5 molar equivalents of sulfo-cyanine3 NHS ester (Lumibrobe) in PBS adjusted to pH ~8.5 with 0.1 M sodium bicarbonate. Excess dye was removed via extensive 0.9 wt% NaCl dialysis followed by extensive diH2O dialysis using a 3 kDa regenerated cellulose membrane (Repligen) and the purified PLE-cy3 was lyophilized until use. For assembly of LbL films with fluorescently tagged PLE, the PLE layering solution was doped with 33% of PLE-cy3.

*Cell Culture*

OV2944-HM-1 cells were acquired through Riken BRC and were cultured in α-MEM supplemented with 10% FBS and 1% penicillin/streptomycin. Glioma stem cell–derived lines from glioblastoma patients (GCGR-E21, GCGR-E57, GCGR-E34, GCGR-E31, GCGR-E28, GCGR-E13) were obtained from the Pollard and Carragher Laboratories through the Glioma Cellular Genetics Resource at the University of Edinburgh.^[3]^ Glioma cells were cultured in DMEM/HAMS-F12 supplemented with N2, B27, 10% glucose, 1% pen/strep, MEM nonessential amino acids, EGF (10 ng/mL) and FGF (10 ng/mL). Culture vessels were coated with Cultrex Laminin (R&D Systems) for 3 h at 10 μg/mL before use. Laminin was added to cell culture media at a concentration of 2 μg/mL. Media was replaced twice weekly and passaged every 5–7 days at a ratio between 1:4 and 1:6 using Accutase (Sigma). Cells were incubated in a 5% carbon dioxide humidified atmosphere at 37 °C. All cell lines were murine pathogen tested and confirmed mycoplasma negative by Lonza MycoAlert™ Mycoplasma Detection Kit.

*In vitro cellular association*

HM-1 cells were plated on a tissue-culture 96-well plate at a density of 50K cells per well. The next day, wells were dosed with NPs and left for the target incubation time. For assessment of NP-associated fluorescence in a fluorescence plate reader, the supernatant was removed from the well and diluted 10X with DMSO. Cells were then washed three times with PBS and disrupted with DMSO. The fluorescence of the NPs associated with cells was then normalized to supernatant fluorescence. The relative fluorescence of each formulation was then compared to an unlayered liposome control containing the same fluorophore.

For analysis via flow cytometry, NPs were dosed at the indicated concentrations and allowed to incubate with cells at 37°C for specific number of hrs in each experiment. Cells were washed with PBS then detached from the plates using 0.25% trypsin and stained with DAPI (1 µg/mL in FACS buffer, 5 min incubation) for viability assessment and fixed with 2% paraformaldehyde (30 min incubation) until analysis by flow cytometry using an LSR Fortessa (BD Biosciences). The EC_50_ of NP binding was determined based on a dose-response curve fit (Hill equation) of the percentage of NP-positive cells for each NP concentration. To estimate the number of glutamate residues per particle, we used the weight equivalents (wt. eq.) of PLE polymers required to reach the zeta potential plateau onset point (POP)^[2]^, together with particle size and density data. For liposomes, an average lipid molecular weight of 800 Da was assumed and the total lipid per liposomes was estimated abased on the surface area of a unilamellar liposome with a lipid headgroup size of 0.71 nm^2^. For polystyrene particles, their mass was estimated based on a sphere volume with a polystyrene density of 1 g/cm^3^.

For experiments dosed with inhibitors, 15 min prior to NP dosing, cells were treated with DMSO or inhibitor diluted 100x into cell media. For Ab blocking experiments, cells were treated with antibodies at a concentration of 5 µg/mL for 1 hr prior to NP addition. For evaluation of extracellular IL-12, after NP dosing and detachment, cells were treated with anti-IL-12 monoclonal Ab for 1 hr at 4 °C, then washed again and fixed for flow cytometry analysis. A general gating strategy is shown in **Figure S10**.

*siRNA depletion*

Knockdown of siRNAs was performed by plating HM-1 cells at 5k cells/well in a 96-well plate. The next day, cells were dosed with anti-SLC1A5 siRNA (ON-TARGETplus siRNA mouse Slc1a5, Horizon Discovery) or scramble siRNA (ON-TARGETplus Non-targeting Control Pool, Horizon Discovery) at 200 nM using Lipofectamine RNAiMAX (ThermoFihser) in Opti-MEM (ThermoFisher) medium according to manufacturer instructions. Four days after siRNA dosing, cells were washed with fresh HM-1 media and dosed with 10 µg/mL of NPs for 30 min prior to cell processing for flow cytometry.

*NanoPrism dataset analysis*

We rank-ordered PRISM cells based on their weighted average NP-cell association after incubation with NPs for 24 hrs as measured in the previously published NanoPrism screen.^[4]^ This metric is indicative of NP association with a cancer cell.^[4]^ The top 100 and bottom 100 cell lines were then compared for differential gene expression within each NP group (i.e., top 100 PLE-NP associated lines compared to the bottom 100 PLE-NP associated lines), and the log-fold change in gene expression and p-value for each gene were generated using the OmicsExpressionProteinCodingGenesTPMLogp1 dataset from DepMap (Public 25Q2) and DESeq2 package in R.^[5]^ For each NP formulation evaluated, this information was used to generate a rank ordered list (calculated as sign(log_2_(foldchange))*-log_10_(p_value_)) to then perform gene set enrichment analysis (GSEA) with the Hallmark Gene Sets.^[6,7]^ The normalized enrichment scores were used for visualization (FDR q value < 0.05), and only Hallmark Gene Sets with significant enrichment in at least one of the three NP groups were shown.

*Confocal Microscopy*

For confocal imaging, 8-well chambered coverglasses (Nunc Lab-Tek II, Thermo Scientific) were coated with rat tail collagen type I (Sigma-Aldrich) per the manufacturer’s instructions. HM-1 cells were plated into the wells at a density of 10K/well and left to adhere overnight prior to NP treatment. After the desired incubation time with NPs, cells were washed 3x with PBS. After washing, cells were fixed in 4% paraformaldehyde for 15 min then washed (3x with PBS) and stained. For cell membrane staining, wheat germ agglutinin (WGA) conjugated to Alexa Fluor488 (Invitrogen) was used. For cell membrane receptor staining, cells were permeabilized for 10 min with 0.1% saponin (Sigma), washed (3x PBS), and then incubated with primary antibodies directed at intracellular epitopes of SLC1A5 or GLUT-1 in 0.5% BSA for at least 3 hrs at 25 °C. Cells were then washed (3x PBS) and stained with secondary anti-rabbit IgG conjugated to Alexa Fluor488 (Invitrogen) for 30 min (for anti-CD44 staining, anti-rat IgG AlexaFluor488 was used). Hoechst 33342 (Thermo Scientific) nuclear staining was included in all preparations following manufacturer’s instructions. Images were analyzed using ImageJ. Slides were imaged on an Olympus FV1200 Laser Scanning Confocal Microscope.

Confocal images of HM-1 cells at a 60x magnification were analyzed on ImageJ using the Correlation Threshold function to determine the correlation between receptor staining and NP fluorescence. Each point represents a single cell.

For evaluation of the percentage of NP signal colocalized on the surface of HM-1 cells, an RO1 on the cell was used and the same function was applied to determine the Mander’s Coefficient between the NP signal and the wheat germ agglutinin cell membrane stain.

*AlphaFold 3 Artificial Intelligence Modeling*

The AlphaFold Server running AlphaFold 3 was used to predict the interaction of four residue-long polypeptides with the human protein sequences of amino acid transporters. Four sodium ions were included in the model. The highest-ranking model predicted for each job in which the polypeptide was docket to the outward-facing binding pocket was used. The highest overall ranking prediction was used if none of the five predictions yielded attachment to the binding pockets. Each amino acid transporter pair was modeled five times and the output inter-chain ipTM between the polypeptide and the transporter was extracted from the model result.

*Ovarian cancer cell line NP association screen*

Previously published data^[8]^ on the association of fluorescently labeled unlayered and layered carboxy-modified latex nanoparticles (NPs) with 10 human ovarian cancer cell lines and 4 healthy primary murine cell types were used for subsequent analyses. To avoid confounding effects from biologically quiescent populations without appreciable endocytic or surface transporter activity lymphocytes (B- and T-cells) were not included. In the prior study, cells were exposed to NPs for 24 hrs, and NP association was quantified by flow cytometry. Z-scores were calculated for each NP formulation, reflecting the relative preference of each cell line for a given NP based on outer-layer polymer identity. In the present work, we assessed the correlation between target gene expression and the fold change in cell-associated median fluorescence intensity (MFI) of PLE- and PLD-coated NPs relative to unlayered NPs. In addition, previously reported Z-scores and the difference in MFI between PLD- and PLE-coated NPs were correlated with gene expression profiles from human ovarian cancer cell lines and healthy primary cells, using single-cell RNA sequencing data from the Human Protein Atlas.^[9]^

*Glioblastoma cell line NP association screen*

Glioblastoma cell lines (GCGR-E21, GCGR-E57, GCGR-E34, GCGR-E31, GCGR-E28, GCGR-E13), were seeded on 96-well plates at a density of 15k cells per well in 100 μL of the appropriate culture media and allowed to adhere overnight. Fluorescent LbL-NPs and UL-NPs generated as described previously were dosed at 5 µg/mL.^[10]^ After 24 hrs of incubation, cells were washed three times with PBS and detached with 25 μL of trypsin-EDTA or Accutase. 200 μL of FACS buffer (PBS with 1% bovine serum albumin and 1 mM EDTA) with 1 μg/mL propidium iodide (Thermo Fisher) was used to quench the dissociation, and cells were pipetted vigorously to achieve a single-cell suspension. Cells were transferred to a new 96-well plate without any laminin or PLL coating, and samples were analyzed using a BD LSR II Flow Cytometer. Gene expression data was provided by the Glioma Cellular Genetics Resource

*Statistical Analysis*

GraphPad PRISM 10 was used to perform statistical analyses. Comparisons between two groups was performed via unpaired t-tests. For multiple groups or multiple variable analysis, one-way, or two-way ANOVAs were used with Tukey’s posthoc correction for time-based analysis or Sidak posthoc for other ANOVA analysis.

Methods References:

[1] G. J. Lieschke, P. K. Rao, M. K. Gately, R. C. Mulligan, “Bioactive murine and human interleukin-12 fusion proteins which retain antitumor activity in vivo” *Nat Biotechnol* **1997**, *15*, 35–40.

[2] I. S. Pires, E. Gordon, H. Suh, D. J. Irvine, P. T. Hammond, “High‐Throughput Microfluidic‐Mediated Assembly of Layer‐By‐Layer Nanoparticles” *Adv Funct Mater* **2025**, DOI 10.1002/adfm.202503965.

[3] S. M. Pollard, K. Yoshikawa, I. D. Clarke, D. Danovi, S. Stricker, R. Russell, J. Bayani, R. Head, M. Lee, M. Bernstein, J. A. Squire, A. Smith, P. Dirks, “Glioma Stem Cell Lines Expanded in Adherent Culture Have Tumor-Specific Phenotypes and Are Suitable for Chemical and Genetic Screens” *Cell Stem Cell* **2009**, *4*, 568–580.

[4] N. Boehnke, J. P. Straehla, H. C. Safford, M. Kocak, M. G. Rees, M. Ronan, D. Rosenberg, C. H. Adelmann, R. R. Chivukula, N. Nabar, A. G. Berger, N. G. Lamson, J. H. Cheah, H. Li, J. A. Roth, A. N. Koehler, P. T. Hammond, “Massively parallel pooled screening reveals genomic determinants of nanoparticle delivery” *Science (1979)* **2022**, *377*, DOI 10.1126/science.abm5551.

[5] Broad, “DepMap: The Cancer Dependency Map Project at Broad Institute,” can be found under depmap.org(accessed 2 July 2025), **2025**.

[6] A. Liberzon, C. Birger, H. Thorvaldsdóttir, M. Ghandi, J. P. Mesirov, P. Tamayo, “The Molecular Signatures Database Hallmark Gene Set Collection” *Cell Syst* **2015**, *1*, 417–425.

[7] Y. Liao, J. Wang, E. J. Jaehnig, Z. Shi, B. Zhang, “WebGestalt 2019: gene set analysis toolkit with revamped UIs and APIs” *Nucleic Acids Res* **2019**, *47*, W199–W205.

[8] S. Correa, N. Boehnke, A. E. Barberio, E. Deiss-Yehiely, A. Shi, B. Oberlton, S. G. Smith, I. Zervantonakis, E. C. Dreaden, P. T. Hammond, “Tuning Nanoparticle Interactions with Ovarian Cancer through Layer-by-Layer Modification of Surface Chemistry” *ACS Nano* **2020**, *14*, 2224–2237.

[9] H. Jin, C. Zhang, M. Zwahlen, K. von Feilitzen, M. Karlsson, M. Shi, M. Yuan, X. Song, X. Li, H. Yang, H. Turkez, L. Fagerberg, M. Uhlén, A. Mardinoglu, “Systematic transcriptional analysis of human cell lines for gene expression landscape and tumor representation” *Nat Commun* **2023**, *14*, 5417.

[10] A. J. Pickering, N. G. Lamson, M. H. Marand, W. Hwang, J. P. Straehla, P. T. Hammond, “Layer-by-Layer Polymer Functionalization Improves Nanoparticle Penetration and Glioblastoma Targeting in the Brain” *ACS Nano* **2023**, DOI 10.1021/acsnano.3c09273.

**Supplemental Figures**


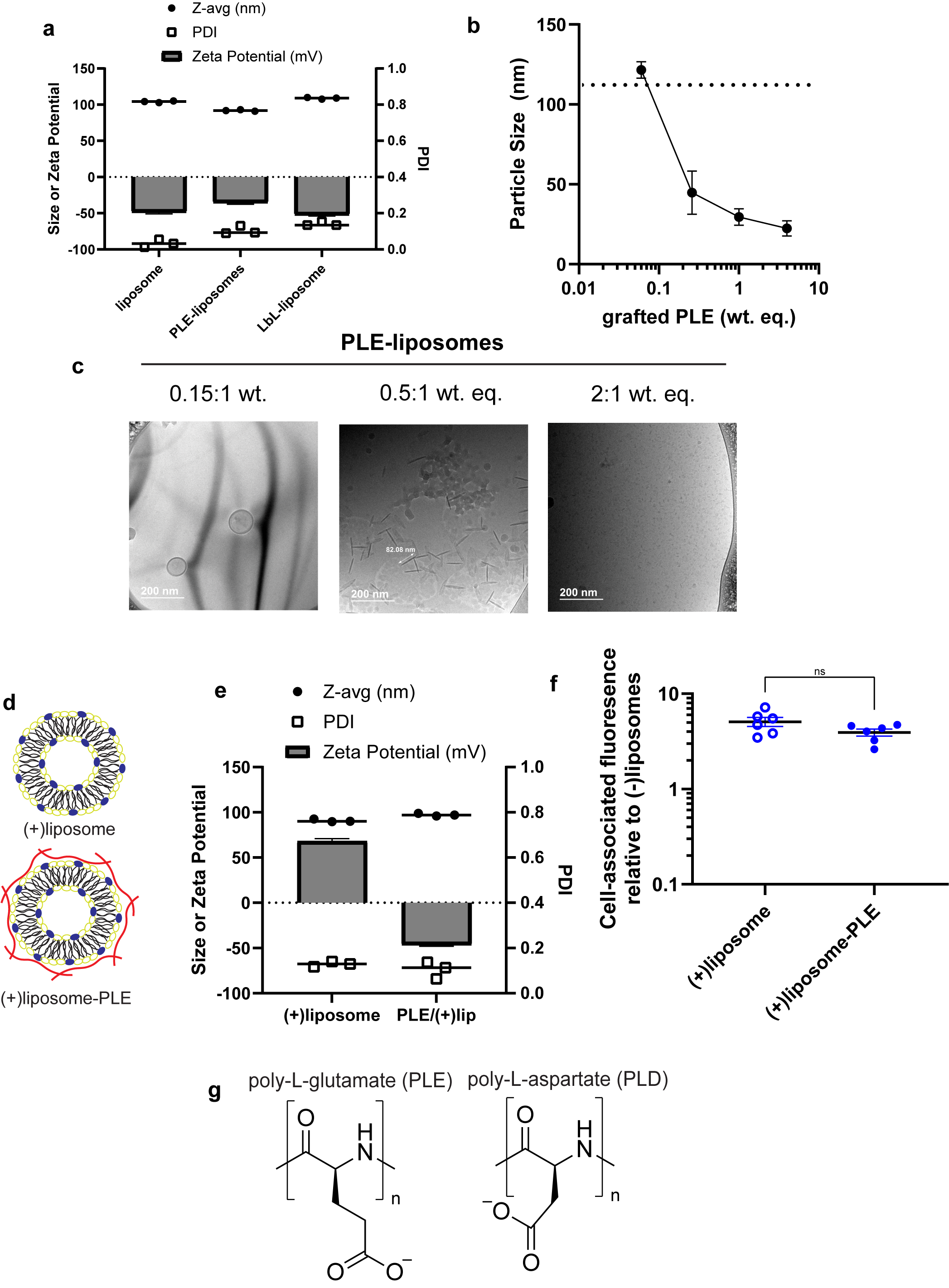


**Figure S1. Characterization of varied NP surface PLE presentation.** (**a**) Size (Z-avg), polydispersity index (PDI), and zeta potential (mean ± s.d.) of standard anionic unlayered liposomes, PLE-grafted liposomes (PLE-liposomes), and anionic liposomes coated with bilayer of PLE and PLR (LbL-liposomes). (**b**) Particle size upon assembly of PLE-liposomes with varied wt.eq. of PLE grafted to lipid. (**c**) Representative cryo-TEM micrograph of assembled NPs with increasing weight equivalents of grafted PLE. (**d-e**) Cationic liposomes were generated and layered with PLE and evaluated for association with HM-1 cells. Shown are the schematic (**d**) and size (Z-avg), PDI, and zeta potential (**e**). (**f**) Fluorescently labeled NPs dosed at 1 µg/mL in HM-1 cells, washed after 4 hr incubation and NP fluorescence associated with cells then measured on plate reader. (**g**) Chemical structure of PLE and PLD. Statistical comparison in **f** was performed via unpaired t-test.


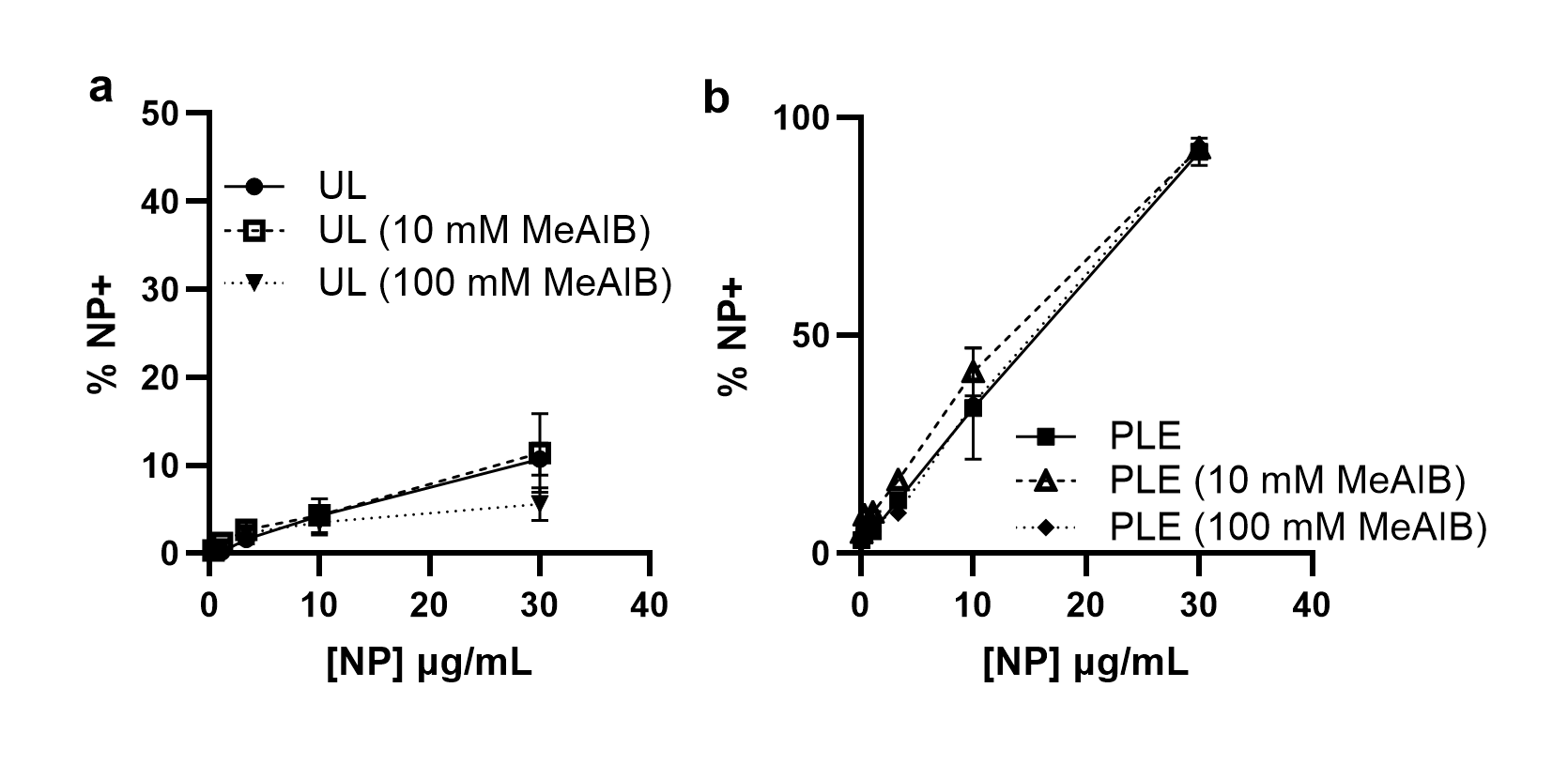


**Figure S2. System A amino acid transport inhibitor MeAIB does not impair PLE-NP binding.** (**a-b**) HM-1 cells were plated in 96 well plates at 50 k cells/well and left to adhere overnight. Cells were then treated with 10 or 100 mM of MeAIB for 15 minutes prior to NP dosing at varying concentrations. Two hours after NP treatment, cells were washed with PBS, and suspended for flow cytometry analysis of NP uptake. Shown are the percentage of PLE- (a) and UL (b) NP+ cells at each concentration of NP dosed.


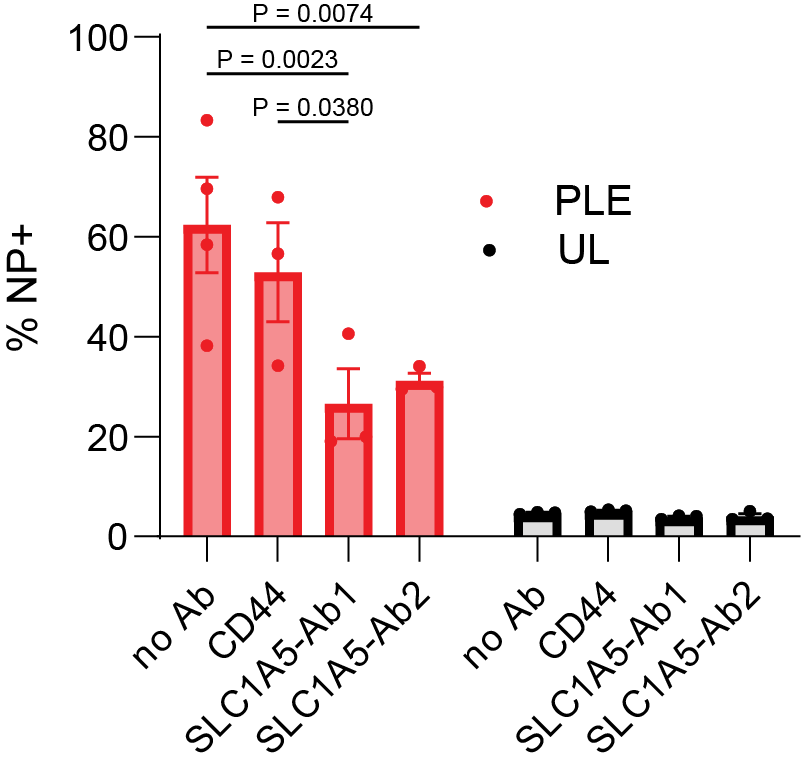


**Figure S3. Ab blockade of PLE-NP association is specific to anti-SLC1A5.** HM-1 cells were treated with antibodies (Abs) against CD44 or two clones against SLC1A5 for 1 hr. Fluorescent UL, PLE, or HA NPs (10 µg/mL) were added for 15 minutes, then cells were washed and analyzed by flow cytometry. Shown are the percentage of NP+ cells for each treatment


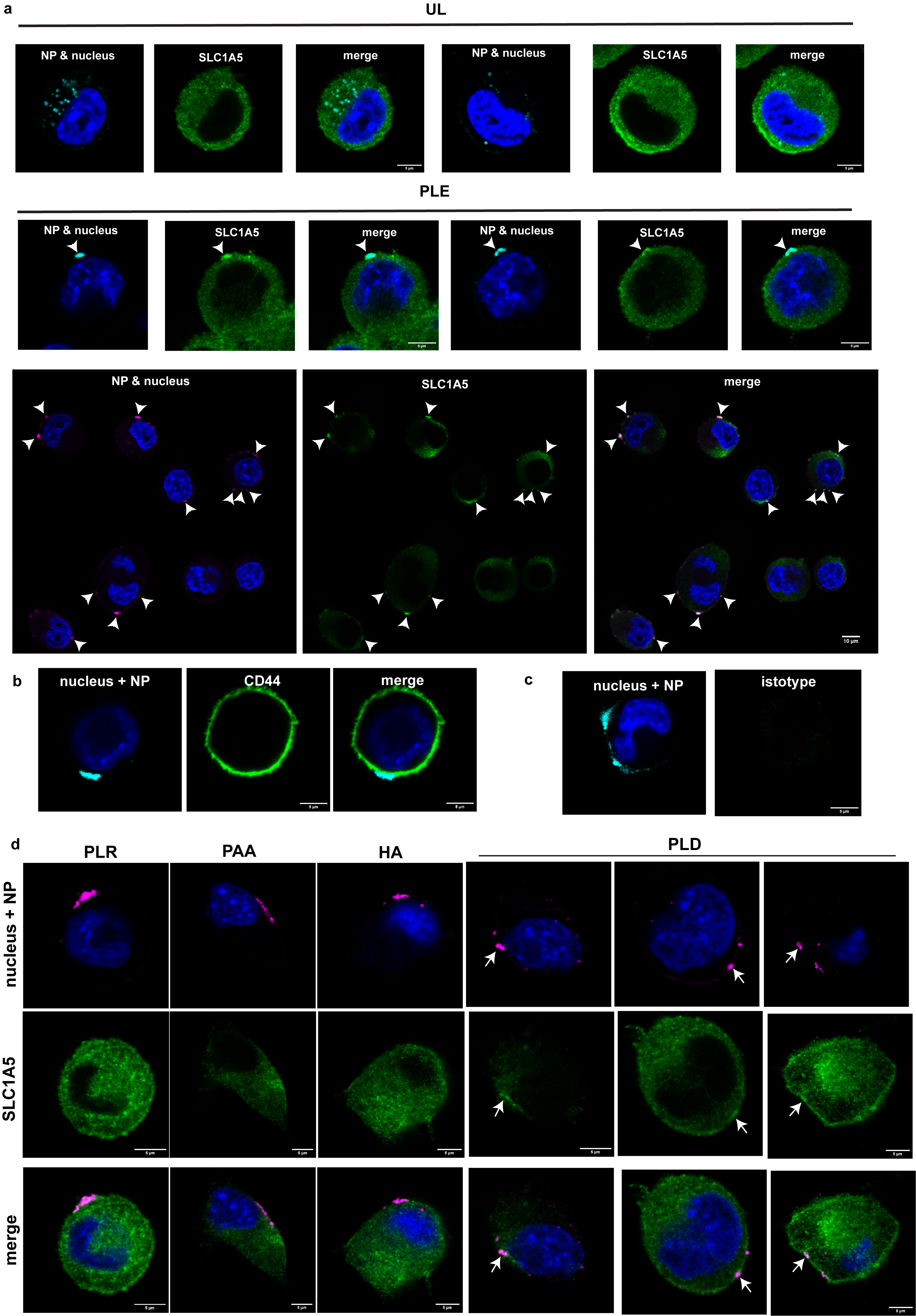


**Figure S4. PLE coating is required for high colocalization of SLC1A5 with LbL-NPs.** (a-d) HM-1 cells were plated in 8-well glass chamber slides at 10 k cells/well and left to adhere overnight. Cells were dosed with 1.5 µg/mL of NPs for 2 hrs. After NP treatment, cells were washed with PBS, fixed with PFA, and then rapidly permeabilized with saponin. Cells were then treated with primary antibodies for 2 hours followed by secondary antibodies for 30 minutes. Shown are HM-1 cells treated with either PLE-NP or UL-NPs and stained with anti-SLC1A5 (a), PLE-NPs and stained with an anti-CD44 Ab (b) or isotype control (c), and HM-1 cells treated with various outer layer LbL-NPs and stained with anti-SLC1A5 Abs (d).


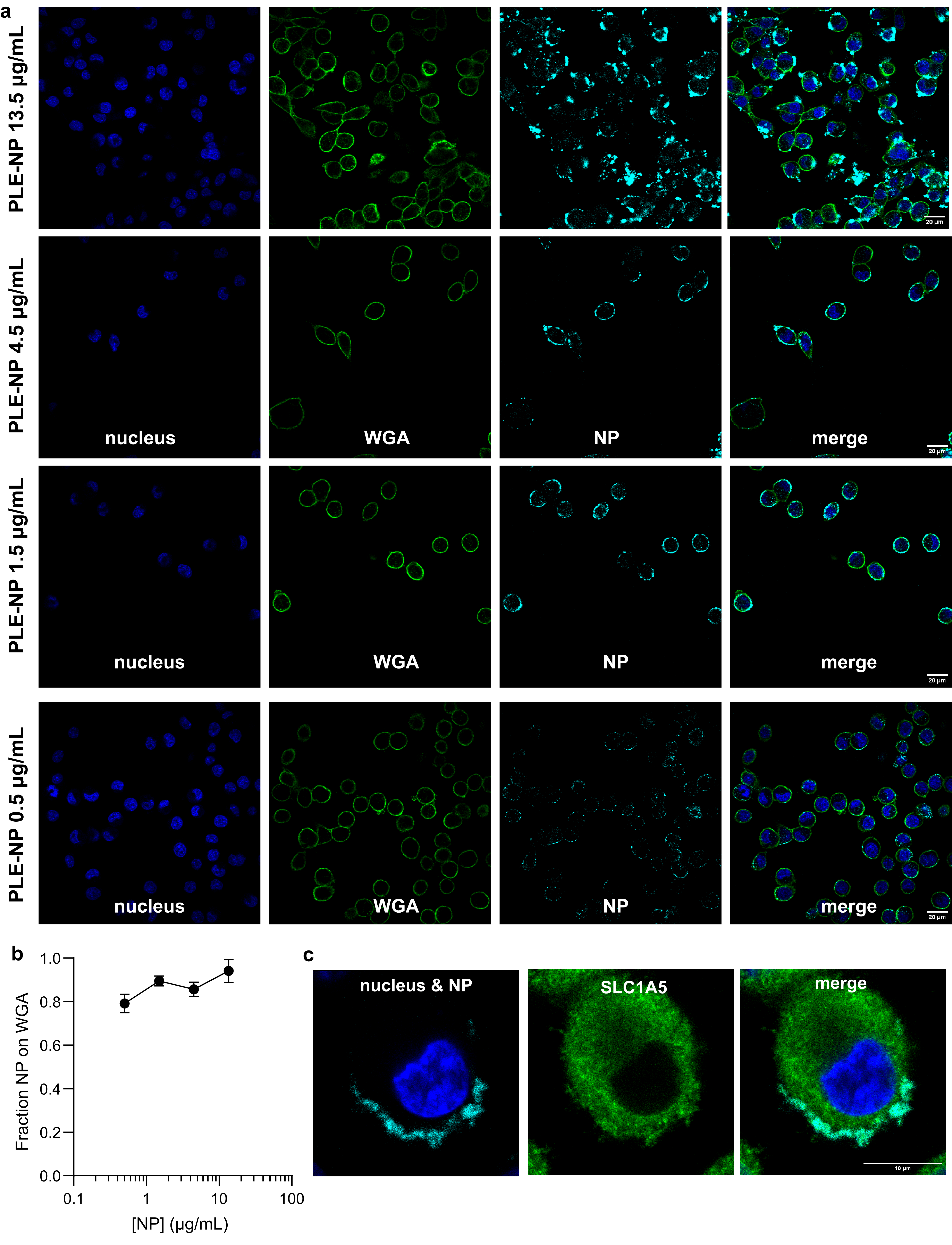


**Figure S5. PLE-NPs associate primarily on the cell membrane.** (a-b) HM-1 cells were plated in 8-well glass chamber slides at 10 k cells/well and left to adhere overnight. Cells were dosed with varying concentrations of NPs for 4 hrs. After NP treatment, cells were washed with PBS, fixed with PFA, and then stained with Hoechst 33342 and wheat germ agglutinin (WGA) and visualized on a confocal microscope. Shown are representative confocal images of HM-1 cells treated with various concentrations of PLE-NPs (a) and quantification of the fraction of NP pixel colocalized with cell membrane pixels (b). (c) HM-1 cells were plated in 8-well glass chamber slides at 10 k cells/well and left to adhere overnight. Cells were dosed with 13.5 µg/mL of NPs for 2 hrs. After NP treatment, cells were washed with PBS, fixed with PFA, and then rapidly permeabilized with saponin. Cells were then treated with primary antibodies for 2 hours followed by secondary antibodies for 30 minutes.


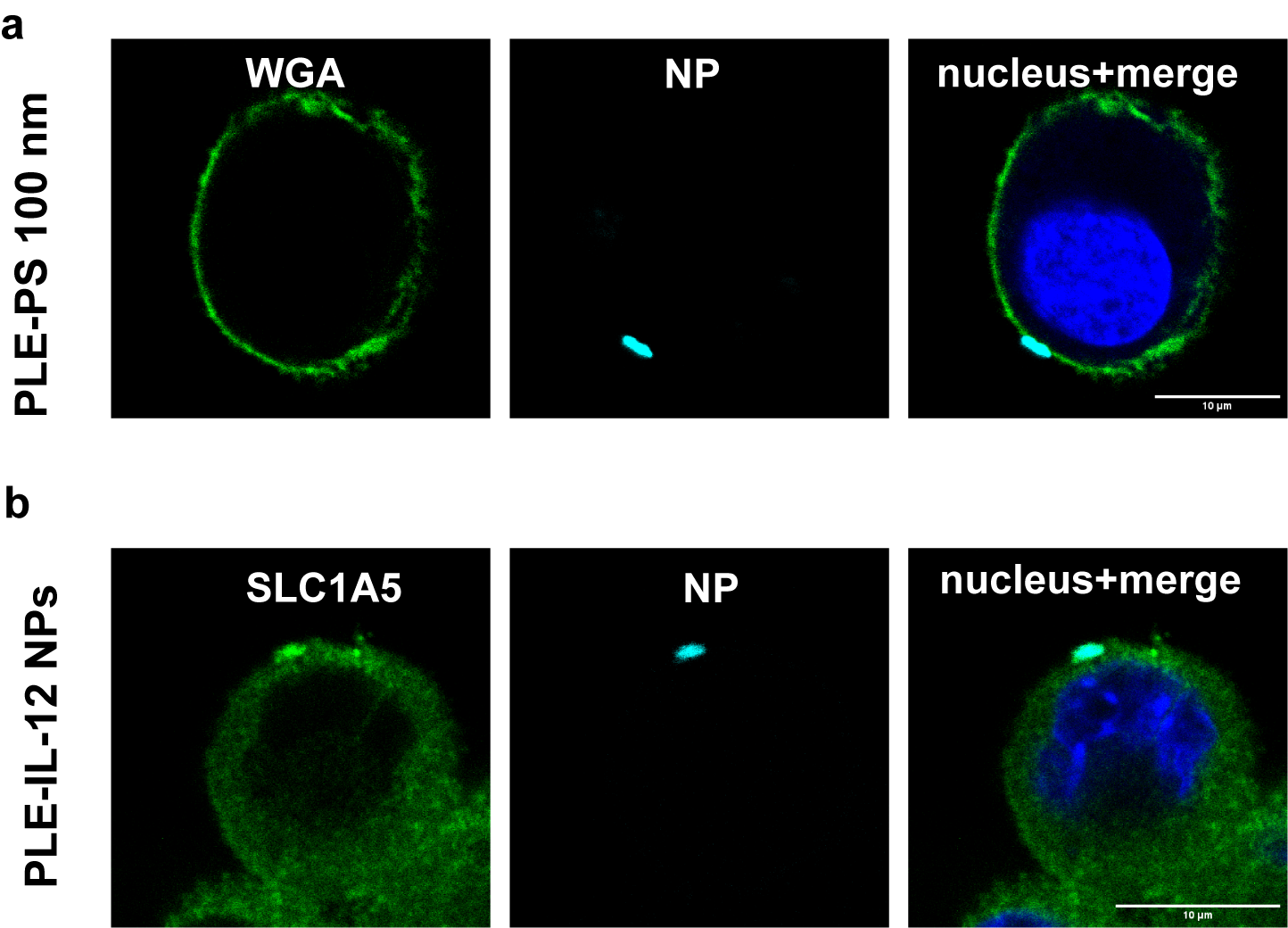


**Figure S6. 100 nm polystyrene PLE-NPs are retained at the cell membrane.** HM-1 cells were plated in 8-well glass chamber slides at 10 k cells/well and left to adhere overnight. Cells were dosed with 1 µg/mL of NPs for 4 hrs. After NP treatment, cells were washed with PBS, fixed with PFA, and then stained with Hoechst 33342 and wheat germ agglutinin (WGA) and visualized on a confocal microscope. Shown is a representative HM-1 cell dosed with 100 nm PLE-PS particles.


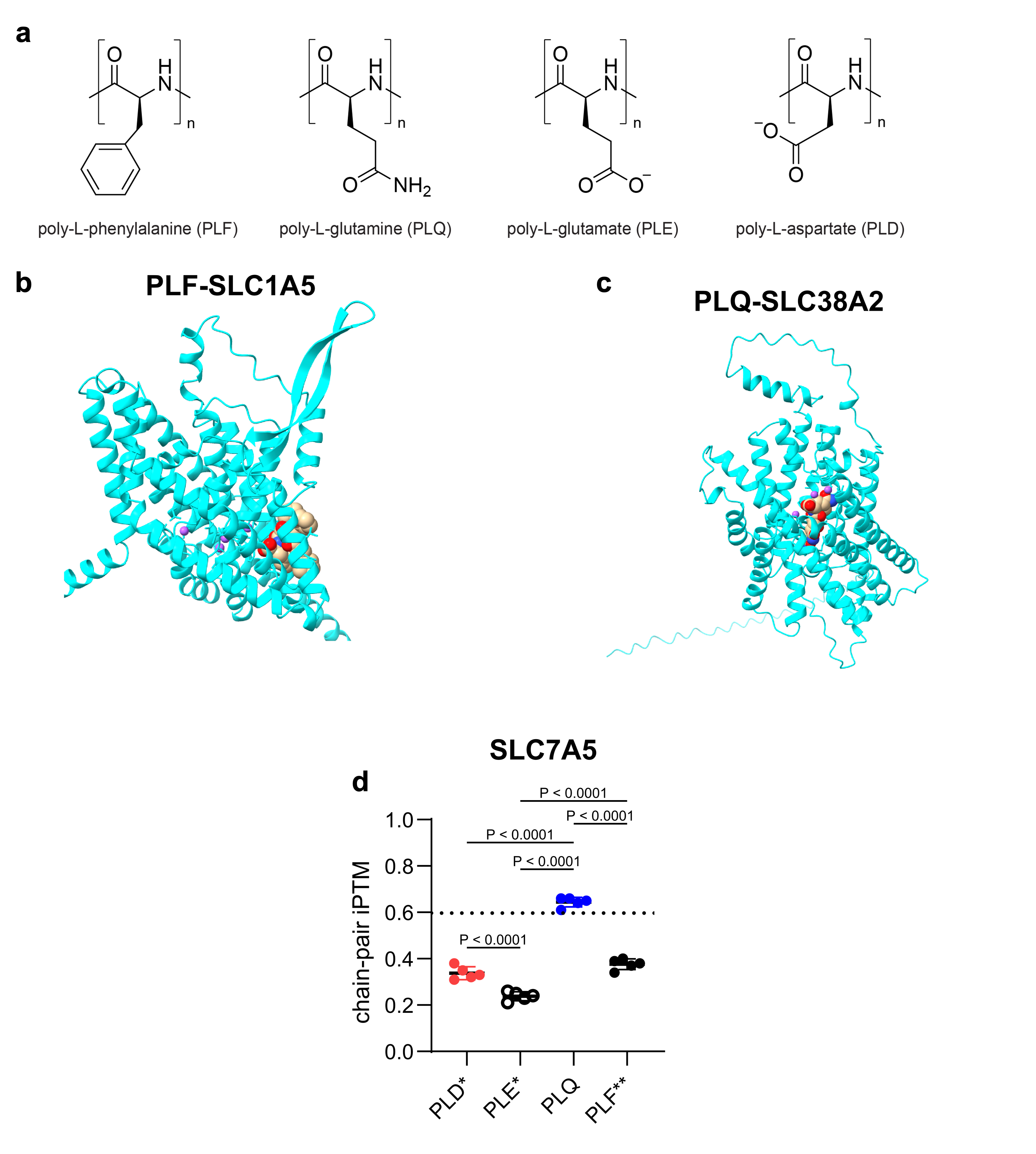


**Figure S7. Modeling predictions.** (a) Chemical structures of polyanions explored in modeling of transporter interactions. (b) Representative AlphaFold 3 model structure of SLC1A5 and PLQ bound to the outward or inward orientation of SLC1A5. (c) Representative AlphaFold 3 model structure of SLC1A5 and PLF binding to a transmembrane region. (d) AlphaFold3 chain pair iPTM scores of modeled PLD, PLE, PLQ, and PLF with SLC7A5 (* indicates polymer not in known binding pocket of transporter).


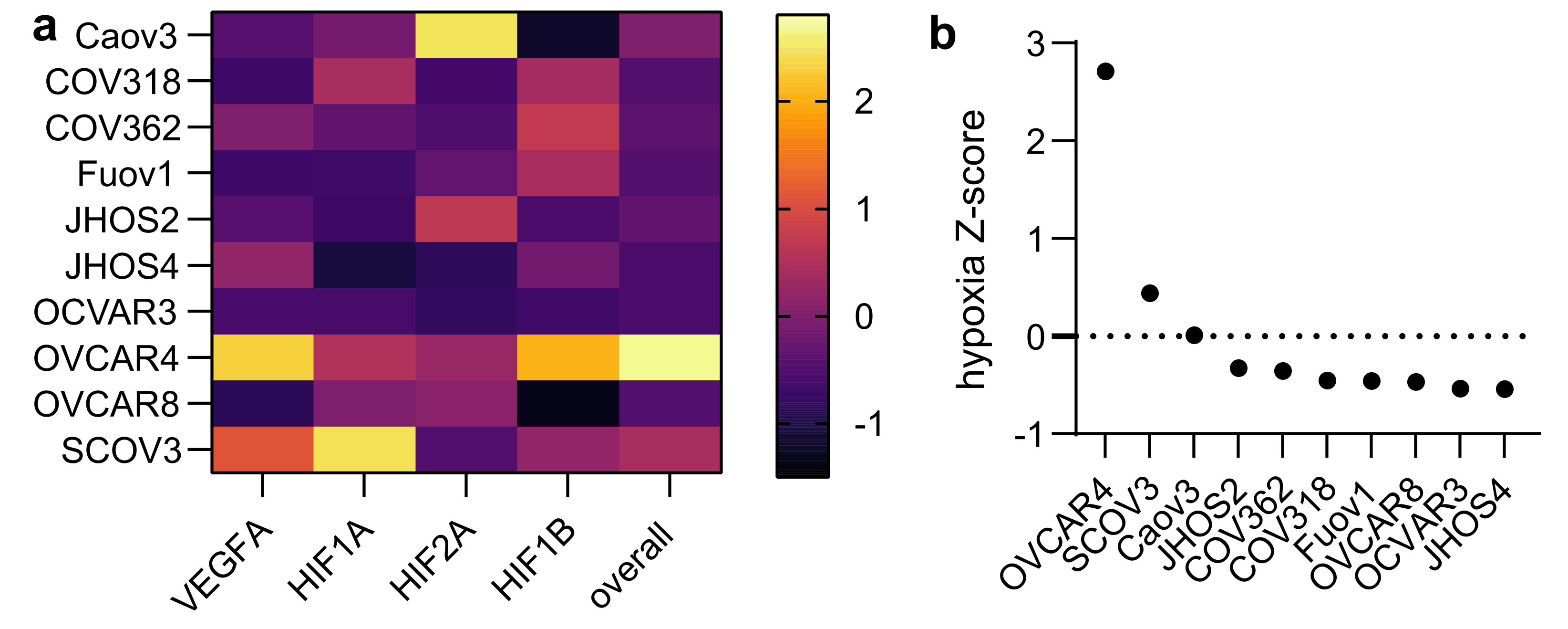


**Figure S8. Analysis of hypoxia-related gene expression in ovarian cancer cell lines.** Heat map of Z-score for each gene across the cell lines and the “overall” hypoxia metric determined by the product of gene expression for each gene.


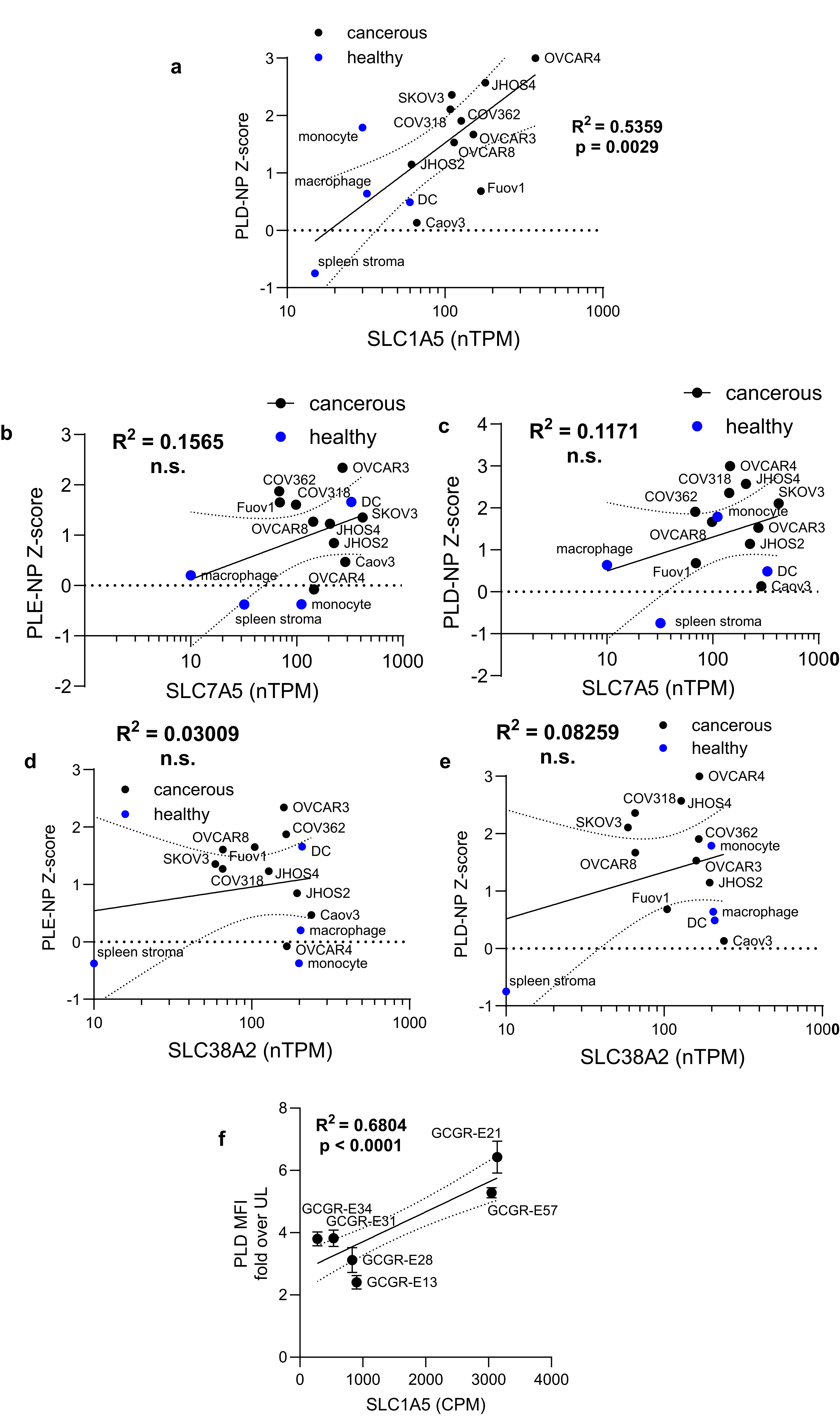


**Figure S9.** (a-e) Analysis of NP association with ovarian cancer cell lines and primary healthy cells with RNA expression of human cell lines derived from Protein Atlas and R^2^ found via linear fit with p-value from non-zero slope test. Dashed lines represent 95% confidence interval of the curve fit. (a) Analysis of PLD-NP Z-scores from NP screen against the same cells as a function of SLC1A5 RNA expression. Analysis of PLE (b, d) and PLD (c, e) Z-scores from NP screen against the same cells as a function of SLC7A5 and SLC38A2 RNA expression. Same analysis as (a-e) but with glioblastoma cell lines and PLD MFI over UL as a function of SLC1A5 mRNA expression.


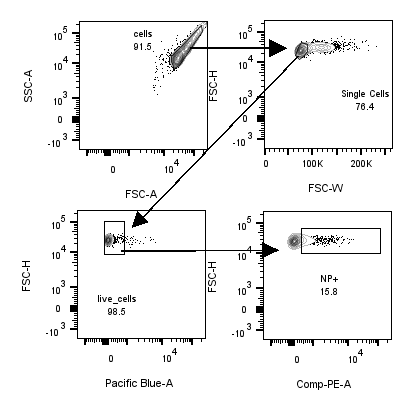


**Figure S10.** Example flow gating strategy.
